# Ac2-26 activated the AKT1/GSK3β pathway to reduce cerebral neurons pyroptosis and improve cerebral function in rats after cardiopulmonary bypass

**DOI:** 10.1186/s12872-024-03909-9

**Published:** 2024-05-21

**Authors:** Ying-nan Ju, Zi-wei Zou, Bao-wei Jia, Zi-ying Liu, Xi-kun Sun, Lin Qiu, Wei Gao

**Affiliations:** 1 Department of Intensive Care Unit, Hainan General Hospital (Hainan Affiliated Hosptial of Hainan Medical University), Clinical College, Hainan Medical University, Haikou, 570311 China; 2 Department of Anesthesiology, The Second Affiliated Hospital of Harbin Medical University, Harbin, Heilongjiang Province 150081 China; 3 Department of Anesthesiology, Hainan General Hospital (Hainan Affiliated Hosptial of Hainan Medical University), Clinical College, Hainan Medical University, Haikou, 570311 China

**Keywords:** Brain injury, Cardiopulmonary bypass, Pyroptosis, Ac2-26

## Abstract

**Background:**

Cardiopulmonary bypass (CPB) results in brain injury, which is primarily caused by inflammation. Ac2-26 protects against ischemic or hemorrhage brain injury. The present study was to explore the effect and mechanism of Ac2-26 on brain injury in CPB rats.

**Methods:**

Forty-eight rats were randomized into sham, CPB, Ac, Ac/AKT1, Ac/GSK3βi and Ac/AKT1/GSK3βa groups. Rats in sham group only received anesthesia and in the other groups received standard CPB surgery. Rats in the sham and CPB groups received saline, and rats in the Ac, Ac/AKT1, Ac/GSK3βi and Ac/AKT1/GSK3βa groups received Ac2-26 immediately after CPB. Rats in the Ac/AKT1, Ac/GSK3βi and Ac/AKT1/GSK3βa groups were injected with shRNA, inhibitor and agonist of GSK3β respectively. The neurological function score, brain edema and histological score were evaluated. The neuronal survival and hippocampal pyroptosis were assessed. The cytokines, activity of NF-κB, S100 calcium-binding protein β(S100β) and neuron-specific enolase (NSE), and oxidative were tested. The NLRP3, cleaved-caspase-1 and cleaved-gadermin D (GSDMD) in the brain were also detected.

**Results:**

Compared to the sham group, all indicators were aggravated in rats that underwent CPB. Compared to the CPB group, Ac2-26 significantly improved neurological scores and brain edema and ameliorated pathological injury. Ac2-26 reduced the local and systemic inflammation, oxidative stress response and promoted neuronal survival. Ac2-26 reduced hippocampal pyroptosis and decreased pyroptotic proteins in brain tissue. The protection of Ac2-26 was notably lessened by shRNA and inhibitor of GSK3β. The agonist of GSK3β recovered the protection of Ac2-26 in presence of shRNA.

**Conclusions:**

Ac2-26 significantly improved neurological function, reduced brain injury via regulating inflammation, oxidative stress response and pyroptosis after CPB. The protective effect of Ac2-26 primarily depended on AKT1/ GSK3β pathway.

**Supplementary Information:**

The online version contains supplementary material available at 10.1186/s12872-024-03909-9.

## Introduction

Cardiopulmonary bypass (CPB) is an essential support for several cardiac surgeries. However, CPB results in severe postoperative cerebral dysfunction [1], which is characterized by stroke [2], cognitive dysfunction [3] and delirium [4]. The incidences of stroke, delirium and cognitive dysfunction are 1.7%-to-5.4% [2], 20%-to-50% [5], and 30%-to-80% [6], respectively. Cerebral dysfunction prolongs the stay in the ICU and hospital, induces physical disability [7], increases medical care costs [8], and increases postoperative mortality [9]. Many studies indicated that local inflammation induced by cerebral ischemia hypoperfusion and systemic inflammatory response syndrome (SIRS) contributed to the postoperative cerebral dysfunction [10, 11]. Although several treatments have been used in clinical work, the incidence of neurological morbidity remain higher and continuously influences the postoperative quality of patients [12].

Ac2-26 is an activated peptide of annexin A1 that mitigates brain injuries by activating formyl peptide receptors [13, 14]. Zhang et al. also found the tripeptide of annexin A1 reduced brain injury after CPB [15], though they did not explore the mechanism. Xu Xin also reported that Annexin A1 protected against cerebral injury induced by ischemia reperfusion via modulating the macrophage polarization [16]. Our previous studies found that Ac2-26 prevented lung and brain reperfusion injury by enhancing the expression of eNOS [17, 18]. These studies prompted us that Annexin A1 and it’s activated peptide (Ac2-26) protected against different organ injury via multiple pathways. Considering the regulation of AKT1 on eNOS, and Akt1/glycogen synthase kinase 3β (GSK3β) pathway in the pathology of brain injury [19], we performed a rat CPB model and administrated the shRNA (knockdown the AKT1) and inhibitor of GSK3β to investigate mechanism of Ac2-26 on brain injury after CPB.

## Methods and materials

The Institutional Animal Care and Use Committee of Harbin Medical University approved this study. Male SD rats (400–450 g) were purchased from Harbin Medical University. All the invasive procedures were performed underwent local anesthesia with lidocaine.

In this study, forty-eight non-repeated numbers were selected from the random number table, and these 48 numbers were allocated to the 48 rats. All the rats were divided into 6 groups according to the assigned number from small to large. The 1st 8 rats which were assigned the smallest 8 numbers were allocated to the sham group, and the 2nd 8 rats were allocated to the CPB group, the 3rd 8 rats were allocated to Ac group, the 4th 8 rats were allocated to Ac/AKT1 group, the 5th 8 rats were allocated into Ac/GSK3βi, and the last 8 rats which assigned the largest 8 number were allocated to Ac/GSK3βa group.

### First section

All the rats were randomized into sham, CPB, Ac, Ac/AKT1 and Ac/GSK3β groups. Rats in the sham group received anesthesia, intubation and catheterization, and the other rats received standard CPB [20]. Rats in the sham and CPB groups were intravenously injected with saline, and rats in the Ac, Ac/AKT1 and Ac/GSK3β groups were injected with Ac2-26. The rats in Ac/AKT1 group were injected the shRNA to knockdown AKT1 expression. Rats in Ac/GSK3β were injected the inhibitor of GSK3β to inhibit the activity of GSK3β. The aim of this section was to preliminary estimate whether the effect of Ac2-26 on brain injury after CPB was associated with AKT1 and GSK3β.

### Second section

In the second section, we administrated the agonist of GSK3β to rats which received shRNA and assessed whether the GSK3β was the down-stream of AKT1 in regulation of Ac2-26 on brain injury.

The neurological function score, histological score, brain permeability, local and systemic inflammation, oxidative stress response, neuronal survival, hippocampal pyroptosis, and pyroptotic protein were evaluated to investigate the mechanism of Ac2-26 on pyroptosis after CPB.

### CPB procedure

The rats were anesthetized with 3% sodium pentobarbital (30 mg/kg). After anesthesia, all of the rats received intubation with a 12-G catheter for mechanical ventilation (Model 683, Harvard Apparatus, Boston, USA). The ventilated parameters were set at 50% oxygen and 50% nitrogen with 2-cm H_2_O positive end-expiratory pressure (Vt: 8 ml/kg, respiratory rate: 50 breaths/min, and inspiratory expiratory ratio 1:1). Under local anesthesia with 1% lidocaine, the right artery and vein and the right femoral artery and vein were catheterized. Heparin (500 IU/kg) was injected for heparinization, and the CPB circuit was inserted. The CPB circuit included a venous reservoir (20 ml), roller pump (Cole Parmer Instrument Company, Chicago, USA) and membrane oxygenator (MeicroPort, Dongguan, Guangdong, China). After priming with 0.2 ml heparin, 11 ml of a hydroethyl starch solution and 0.5 ml 7% sodium bicarbonate solution, the flow rate was gradually up-regulated to 100 ml/kg/min for 60 min [21]. During CPB, the mean arterial pressure was maintained over 70 mmHg with adrenalin, and the temperature was maintained at 36 to 38 ℃. After 60 min of CPB, protamine (5 mg/kg) [22] was injected for anti-heparinization, the outflow was withdrawn first. The inflow was stopped when hemodynamic stabilization was achieved. All catheters were withdrawn, and the incisions were saturated after the injection of 2000 U/kg penicillin to prevent infection. All rats were extubated when they recovered spontaneous breathing.

Immediately after the initiation of CPB, rats in the CPB group received saline (0.5 ml), and rats in other groups were injected with Ac2-26 (1 mg/kg diluted into 0.5 ml) [17, 18]. Rats in the Ac/AKT1 group were intravenously injected with shRNA (1 × 10^5^ ifu/L AKT1 expression lentiviral solution at 150 µL/100 g body weight) to knock down AKT1 expression preoperatively for 3 days according to our preliminary study. The GSK3β inhibitor was injected intraperitoneally (SB216763, 20 mg/kg diluted into 1 ml of DMSO/saline) when CPB beginning [23].

After 12 h of CPB, the neurological function of all rats was tested. All rats were injected with heparin and sacrificed via an overdose of anesthetics [24].

### Neurological function score

Neurological function was scored according to the following criteria: prehensile traction, strength and balance beam performance were graded on a 0–9 scale (best score = 0, worst score = 9). Evaluations of neurological function were performed according to a previous study [15].

### Brain edema

After sacrifice, part of the brain tissue was collected and weighed after removal of the parietal cortex and hippocampus. The brain tissue was dried at 80 °C for 48 h and weighed again. Brain edema was calculated using the (wet weight – dry weight)/wet weight ratio [25].

### Histological evaluation

Brain hippocampal tissue was collected and fixed in neutral formalin. The tissue was dehydrated with ethanol, cleared with xylene, and embedded in paraffin. The brain tissue was cut into 4-µm sections and stained with HE. After staining, an independent pathologist who did not participate in this study assessed pathological injuries using light microscopy. Ten randomized fields of each sample were analyzed to appraise the brain injury.

### Neuronal survival and pyroptosis assessment

Neuronal survival was analyzed using Nissl staining. The brain tissues were fixed with 4% paraformaldehyde and placed on polylysine-coated slides overnight. After rehydration, the sections were submerged in 1% cresyl violet before staining. The surviving neurons of the hippocampal CA1 region exhibited the standard of characteristics of a visible nucleus and intact cytoplasm. An independent pathologist counted the surviving neurons.

The pyroptosis of hippocampus was detected by the immunohistochemistry using the cleaved-gasdermin D (GSDMD) (PU224937, Rabbit anti Rat, 1:500) (Abmart, Shanghai, China). The slide was deparaffinized and hydrated, and the slide was antigen retrieved using the antigen retrieval solution. The endogenous enzyme activity was inactivated by the hydrogen peroxide dropwise, and blocked by the goat serum blocking solution. After blocking, the section was incubated with primary-antibody for 24 h, and then the section was washed. The section was further incubated the horseradish peroxidase-conjugated secondary antibody (sc-2357, Mouse anti Rabbit, 1:500)) and finally colored using DAB chromogenic solution. The cells stained with brown was charged with positive cells. Ten randomized fields of each sample were analyzed to examine the pyroptosis.

### Cytokine measurement in the serum and brain

To observe the effect of Ac2-26 on inflammation induced by PCB, we detected TNF-α (EK0526), IL-1β (EK0393), IL-6 (EK0412), and IL-10 (EK0418) levels (Boster, Wuhan, China) and S100 Calcium-binding protein β (S100β) (E0567r) (EiAab, Wuhan, China) and Neuron-specific enolase (NSE) (R0058) (Elabscience, Wuhan, China) in the serum using commercial ELISA kits. The brain tissue was collected and homogenized in saline (1:9) to prepare brain homogenates. The homogenate was centrifuged at 14,000 × g for 10 min at 4 °C, and the supernatant was collected. All supernatants were tested using ELISA kits according to the manufacturer’s instructions.

### Cerebral oxidative stress response after CPB

To explore the effect of Ac2-26 on oxidative stress response induced by CPB, the concentrations of malondialdehyde (MDA), and activity of myeloperoxidase (MPO) and xanthine oxidase (XO) in brain tissues were tested with a Coomassie blue dye-binding assay (Nanjing Jiancheng Corp, China).

### Western blotting

Brain hippocampal samples were lysed with RIPA buffer. The protein levels in brain tissue were detected using the Bradford method. After confirmation of the protein concentration, equal amounts of protein from each rat were added to the gels and transferred onto polyvinylidene fluoride (PVDF) membranes after electrophoresis. The blots were cut prior to hybridization with antibodies during blotting.After the transfer of proteins, the PVDF membrane was blocked with 5% dry milk and incubated with primary antibodies of AKT1 (#2938, Rabbit anti rat, 1:2000), GSK3β (#9315, Rabbit anti rat, 1:2500), (Cell Signaling Technology, USA), NLRP3 (P60622R3, Rabbit anti rat, 1:1000), cleaved caspase-1 (PY10200, Rabbit, 1:1000), cleaved-GSDMD (PU224937, Rabbit anti rat, 1:2000) (Abmart, Shanghai, China). The incubated membrane was washed with PBS then incubated with a horseradish peroxidase-conjugated antibody (sc-2357, Mouse anti Rabbit, 1:1000) (Santa Cruz Biotechnology, Santa Cruz, USA). The bands on the membrane were visualized with enhanced chemiluminescence developing solutions and quantified using ImageJ software.

### Statistical analysis

In this study, the sample size was determined according to our preliminary study using the primary outcome, neurological score. In our preliminary study, the neurological score in CPB group was 5 ± 0.75, and the neurological score in Ac group was 3.5 ± 0.71. Seven rats were required to detect the difference of 1.5 in neurological score with an alpha error of 0.05 and a power of 90%. To avoid the influence of death of rat on results, we enrolled 8 rats in each group.

The normality of data was tested using Shapro-wilk test. The normally data were presented as the means ± SD. The data were analyzed using analysis of variance (ANOVA) in SPSS 19.0 (SPSS, Chicago, IL, USA). The difference between two groups was calculated using the Bonferroni test. *P* < 0.05 was considered statistically significant.

## Results

### Ac2-26 ameliorated neurological function after CPB

**Fig. 1:**
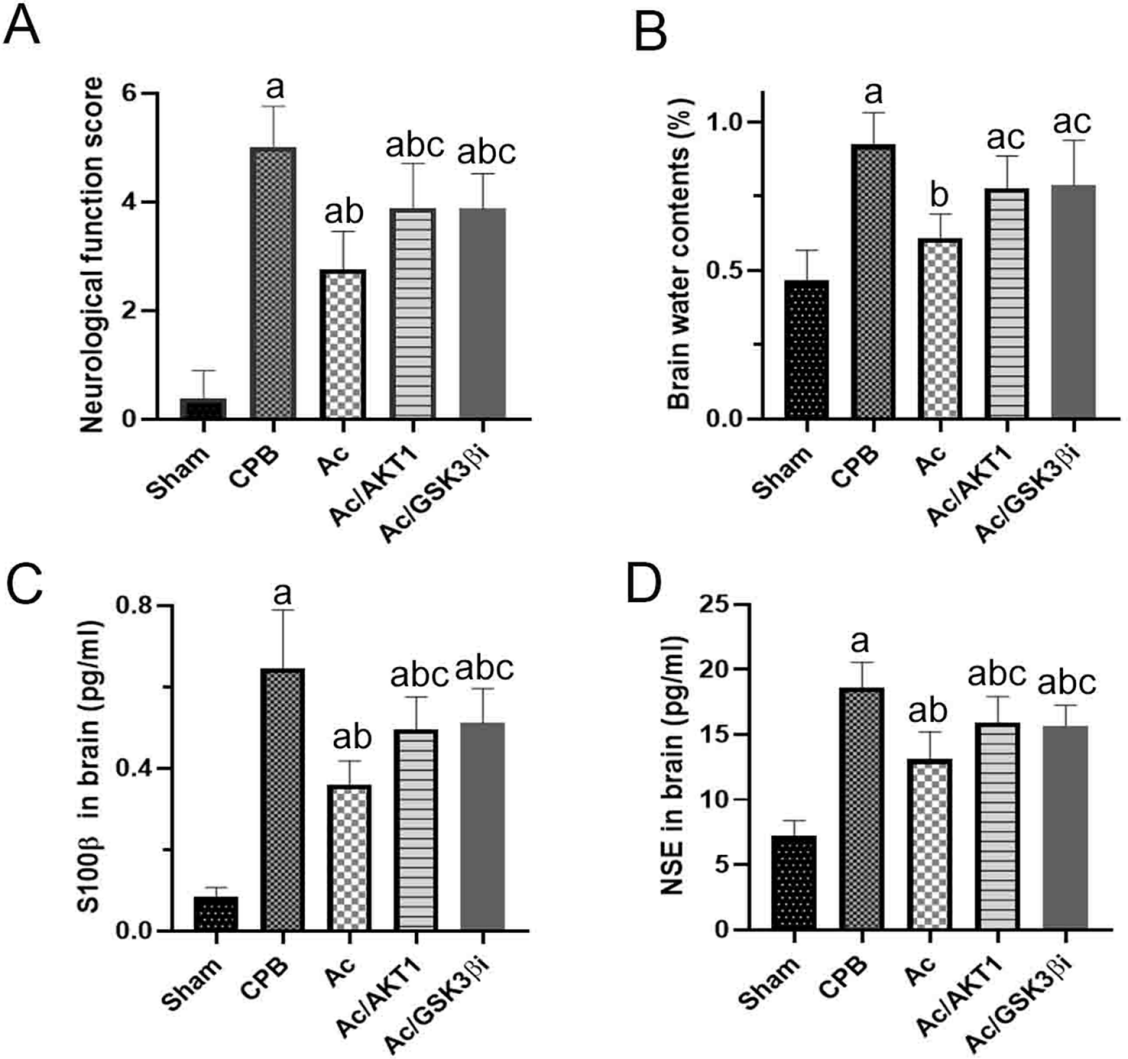
Ac2-26 ameliorated brain injury after CPB. *legends* Compared to the sham group, the neurological score in the CPB group was significantly higher. Compared to CPB, Ac2-26 notably up-regulated the score, and shRNA and inhibitor of GSK3β reduced the effect of Ac2-26 (Fig. 1A). After CPB, the (wet weight – dry weight)/weight ratio notably increased in the CPB group and decreased by Ac2-26 in the Ac group. The shRNA and inhibitor of GSK3β reduced the improvement in brain edema (Fig. 1B). Similar, S100β and NSE increased significantly after CPB, but decreased by Ac2-26. The Ac2-26-induced decrease in S100β and NSE was partially reduced by shRNA and inhibitor of GSK3β (Fig. 1C and D)

Compared to the sham group, the neurological score of rats in the CPB group was significantly higher. Ac2-26 notably down-regulated the neurological score in rats underwent CPB, and the shRNA and inhibitor of GSK3β notably inhibited the improvement of Ac2-26 on neurological score (Fig. 1A).

### Ac2-26 improved the blood‒brain barrier after CPB

After CPB, the (wet weight – dry weight)/weight ratio notably increased in the CPB group and decreased by Ac2-26 in the Ac group. Both the shRNA and inhibitor of GSK3β reduced the improvement of Ac2-26 on brain edema (Fig. 1B).

S100β and NSE levels in the brain were also tested using commercial ELISA kits. After CPB, S100β and NSE significantly increased in the CPB group and decreased by Ac2-26. The Ac2-26-induced decrease in S100β and NSE was partially reduced by shRNA and inhibitor of GSK3β (Fig. 1C and D).

### Ac2-26 mitigated the pathological brain injury

**Fig. 2:**
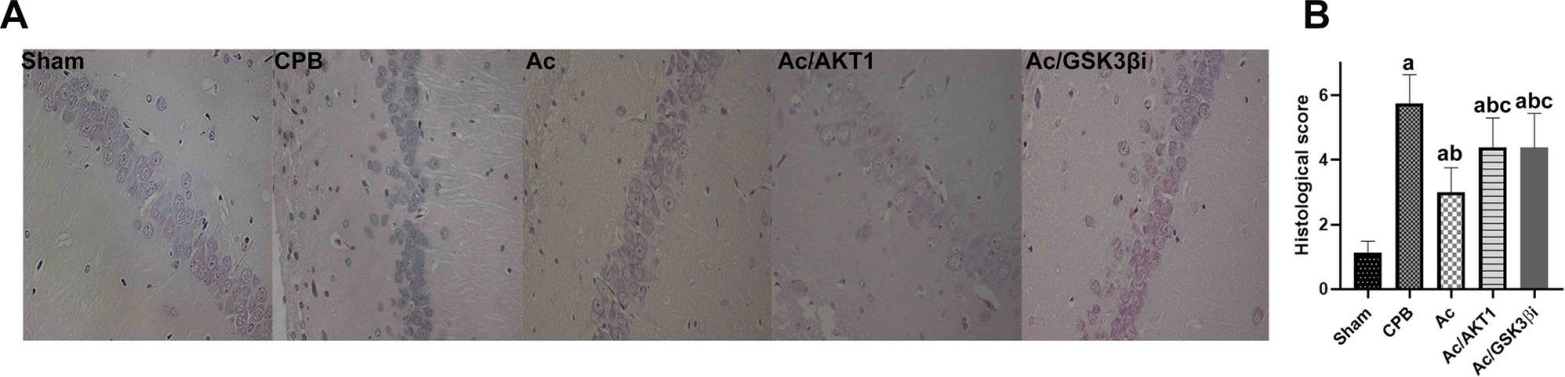
Ac2-26 mitigated the pathological brain injury. *legends* Fig. 2A shows the normal hippocampal neurons, which exhibited clear boundaries and a normal structure in the sham group. Compared to the sham group, Fig. 2B presents typical brain injuries, such as cellular degeneration and abnormal cell arrangements, in the CPB group. Compared to the CPB group, Ac2-26 significantly alleviated the pathological injury in the Ac group, but the shRNA and inhibitor of GSK3β notably reversed the Ac2-26-induced effects (Fig. 3)

Histological analysis shows the normal hippocampal neurons, which exhibited clear boundaries and a normal structure. In sham group, the neuronal cells in hippocampus CA1 region were arranged with regular and orderly, clear and neatly, and stained uniform. After 12 h of CPB, the typical brain injuries, such as cellular degeneration and abnormal cell arrangements, were observed in rats of CPB group. Compared with sham group, the neuronal cells in CPB group were damaged and presented severe disordered with staining ununiform. Compared with CPB group, the arrangement and staining of neuronal cells was improved by Ac2-26, and the improvement of Ac2-26 was attenuated by shRNA and inhibitor of GSK3β(Fig. 2A). The histological score and statistical results were presented in Fig. 2B.

### Ac 2–26 improved neuronal survival

**Fig. 3:**
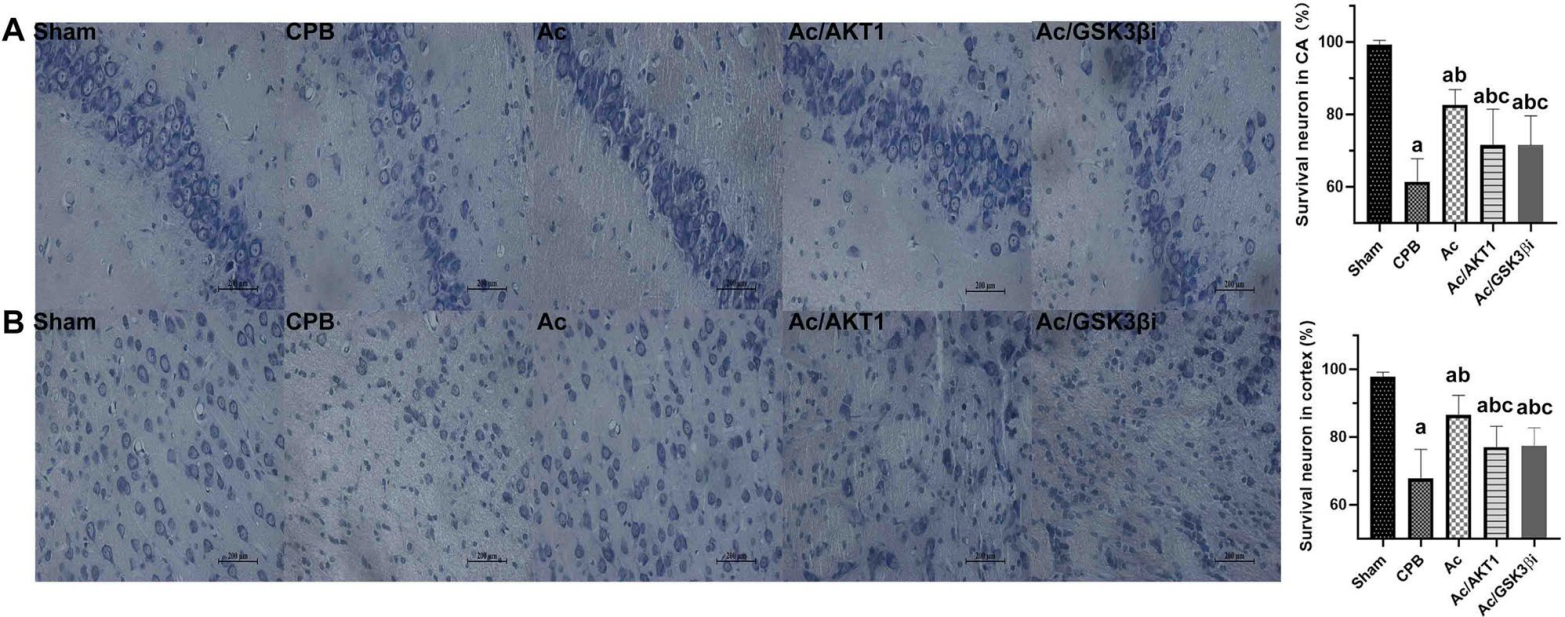
Ac 2–26 improved neuronal survival after CPB. *legends* The survival of neurons in hippocampal and cortex were presented in A and B respectively

The neurons in hippocampal CA1 region and cortex were assessed. Compared to the sham group, neuronal survival in the hippocampal CA1 and cortex region was significantly decreased in the CPB group. Compared to the CPB group, Ac2-26 significantly improved neuronal survival, but the shRNA and inhibitor of GSK3β significantly reduced the Ac2-26-induced improvement in neuronal survival (Fig. 3A and B).

In section A, the neuronal survival in the hippocampal CA1 region was significantly decreased in the CPB group. Compared to the CPB group, Ac2-26 significantly improved neuronal survival, but the shRNA and inhibitor of GSK3β significantly reduced the Ac2-26-induced improvement in neuronal survival.

Similar with section A, the neurons in cortex were also significantly reduced after CPB. The Ac2-26 notably increased the neurons survival rate in rats received Ac2-26 treatment. Nevertheless, the shRNA and inhibitor of GSK3β lessened the biological effect of Ac2-26. The images were presented with 400 magnifications.

### Ac 2–26 reduced pyroptosis of neurons

**Fig. 4:**
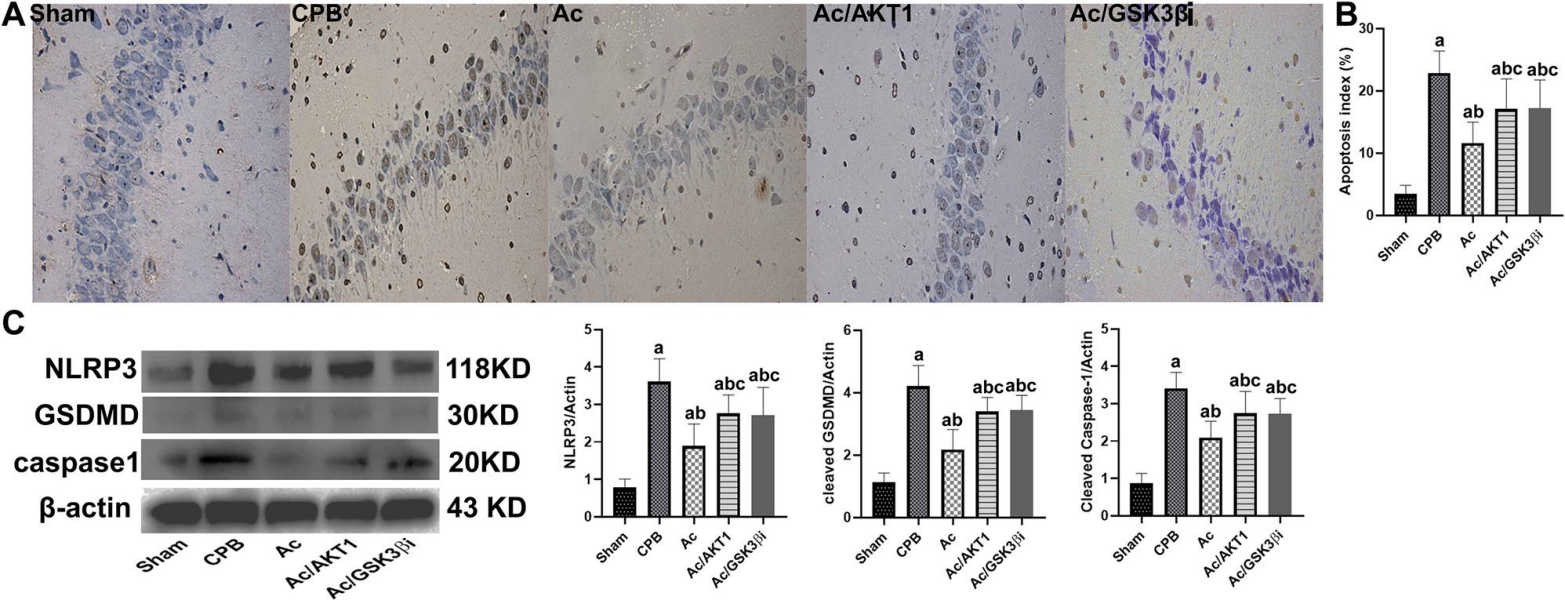
Ac 2–26 reduced the pyroptosis in brain tissue after CPB. *legends* The pyroptosis was evaluated by the immunohistochemistry using cleaved GSDMD antibody (**A**). The positive pyroptotic cells in hippocampus region were increased after induction of CPB, but the Ac2-26 significantly decreased the positive pyroptotic cells. However, the shRNA and inhibitor of GSK3β significantly reduced the Ac2-26-induced anti-pyroptotic effect. The images were presented with 400 magnifications. The pyroptotic index was presented in Fig. 4B

We detected the pyroptosis induced by CPB in brain tissue using immunohistochemistry and western blot. Compared to the sham group, severe pyroptosis in brain tissue were detected after 12 h of CPB. Compared to the CPB group, Ac2-26 significantly decreased the number of positive cells in the hippocampus, but the shRNA and inhibitor of GSK3β partially reversed the reduction of Ac2-26 on pyroptosis (Fig. 4A). The percentage of pyroptosis was presented in Fig. 4B.

We also detected pyroptotic proteins in the brain tissue to further investigate the effect of Ac2-26 on pyroptosis induced by CPB. After CPB, the pyroptotic protein NLRP3, cleaved caspase-1 and cleaved-GSDMD were significantly increased, and the Ac2-26 inhibited the increase of pyroptotic proteins. The inhibition of Ac2-26 on pyroptotic proteins was attenuated by the shRNA and inhibitor of GSK3β (Fig. 4C).

The pyroptotic proteins in brain tissue, including NLRP3, cleaved caspase1 and cleaved GSDMD, were detected (B). After 12 h of CPB, the pro-pyroptotic protein NLRP3, cleaved caspase1 and cleaved GSDMD in brain tissue were up-regulated, but down-regulated by the Ac2-26. Compared with Ac group, the shRNA and inhibitor of GSK3β alleviated the regulation of Ac2-26 on pyroptotic proteins (C).

### Ac2-26 inhibited local inflammation after CPB

**Fig. 5:**
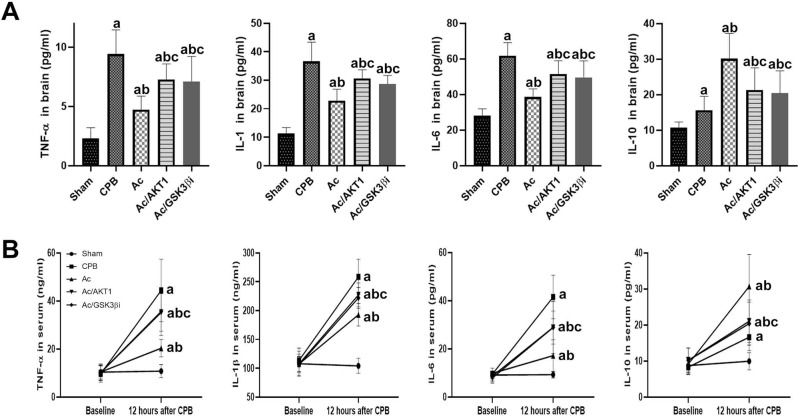
Ac2-26 inhibited local and systemic inflammation after CPB. *legends* After 12 h of CPB, TNF-α, IL-1β, IL-6, and IL-10 were significantly increased in the brain tissue (**A**) and serum (**B**). Ac2-26 reduced TNF-α, IL-1β and IL-6 but upregulated IL-10 in the Ac group. However, the AKT1 RNA interference partially reversed the regulatory effect of Ac2-26 on inflammatory cytokines

After CPB, the levels of TNF-α, IL-1β, IL-6, IL-10 were significantly increased in the brain. These results suggested that severe local inflammation occurred in brain tissue. Compared with sham group, the Ac2-26 notable reduced TNF-α, IL-1β and IL-6 concentrations but upregulated IL-10 in the Ac group. However, the shRNA and inhibitor of GSK3β partially reversed the regulatory effect of Ac2-26 on inflammatory cytokines in brain tissue (Fig. 5A).

Furthermore, we also detected the inflammatory factors in serum to investigate the effect of Ac2-26 on systemic inflammation after CPB. Similar to brain tissue, all the inflammatory factors in serum after CPB significantly increased compared with baseline. The Ac2-26 down-regulated the pro-inflammatory factors, but up-regulated anti-inflammatory cytokines. The regulated effect of Ac2-26 was significantly lessened by the shRNA and inhibitor of GSK3β (Fig. 5B).

### Ac2-26 inhibited cerebral oxidative stress response after CPB

**Fig. 6:**
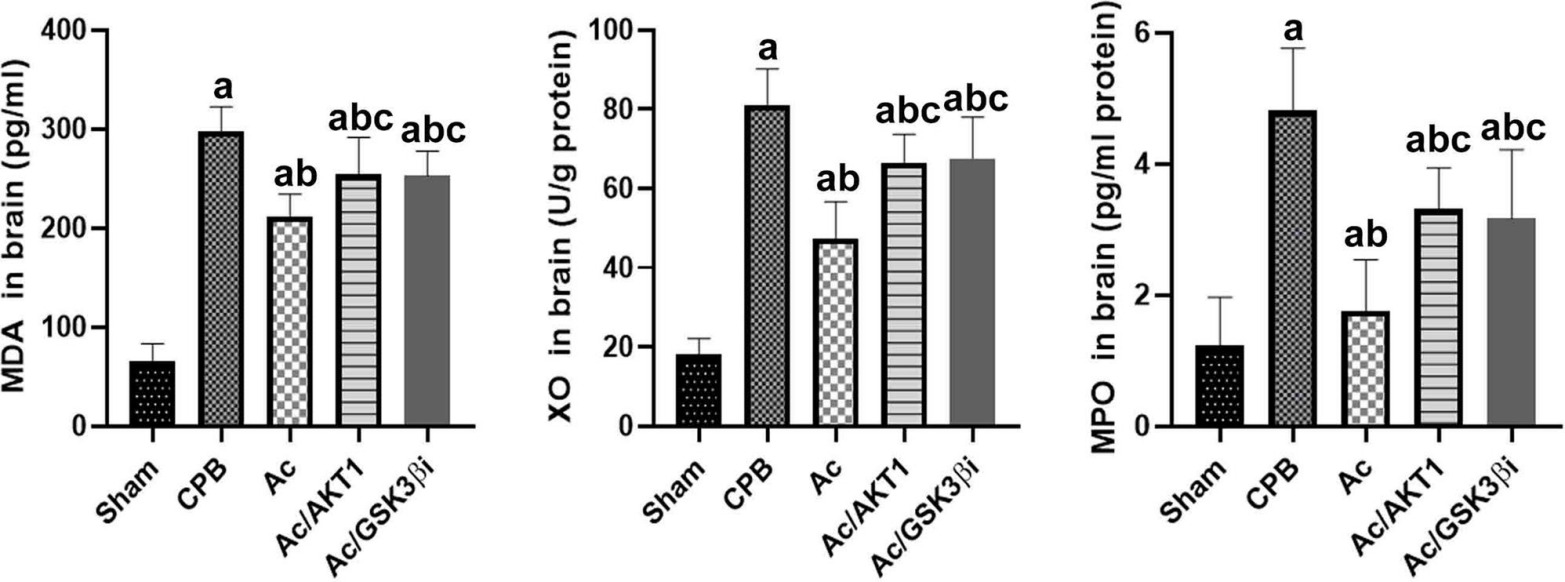
Ac 2–26 inhibited oxidative stress response. *legends* After 12 h of CPB, the MDA levels and activity of MPO and XO in the brain tissues were tested. After CPB, the concentration of MDA was significantly increased in brain tissue. The activity of MPO and XO was also up-regulated in brain tissue. In Ac group, the Ac2-26 notably decreased the levels of MDA and down-regulated the activity of MPO and XO after CPB. The anti-oxidative effect of Ac2-26 was significantly alleviated by the shRNA and inhibitor of GSK3β

After 12 h of CPB, the MDA levels and MPO and XO activation in the brain tissues were tested. After CPB, the concentration of MDA was significantly increased in brain tissue. The activity of MPO and XO was also up-regulated in brain tissue (*P* < 0.05). Compared with CPB group, the Ac2-26 notably decreased the levels of MDA and down-regulated the activity of MPO and XO after CPB (*P* < 0.05). The anti-oxidative effect of Ac2-26 was significantly alleviated by the shRNA and inhibitor of GSK3β (Fig. 6).

### Ac2-26 reduced the brain injury depended on AKT1/GSK3β pathway

**Fig. 7:**
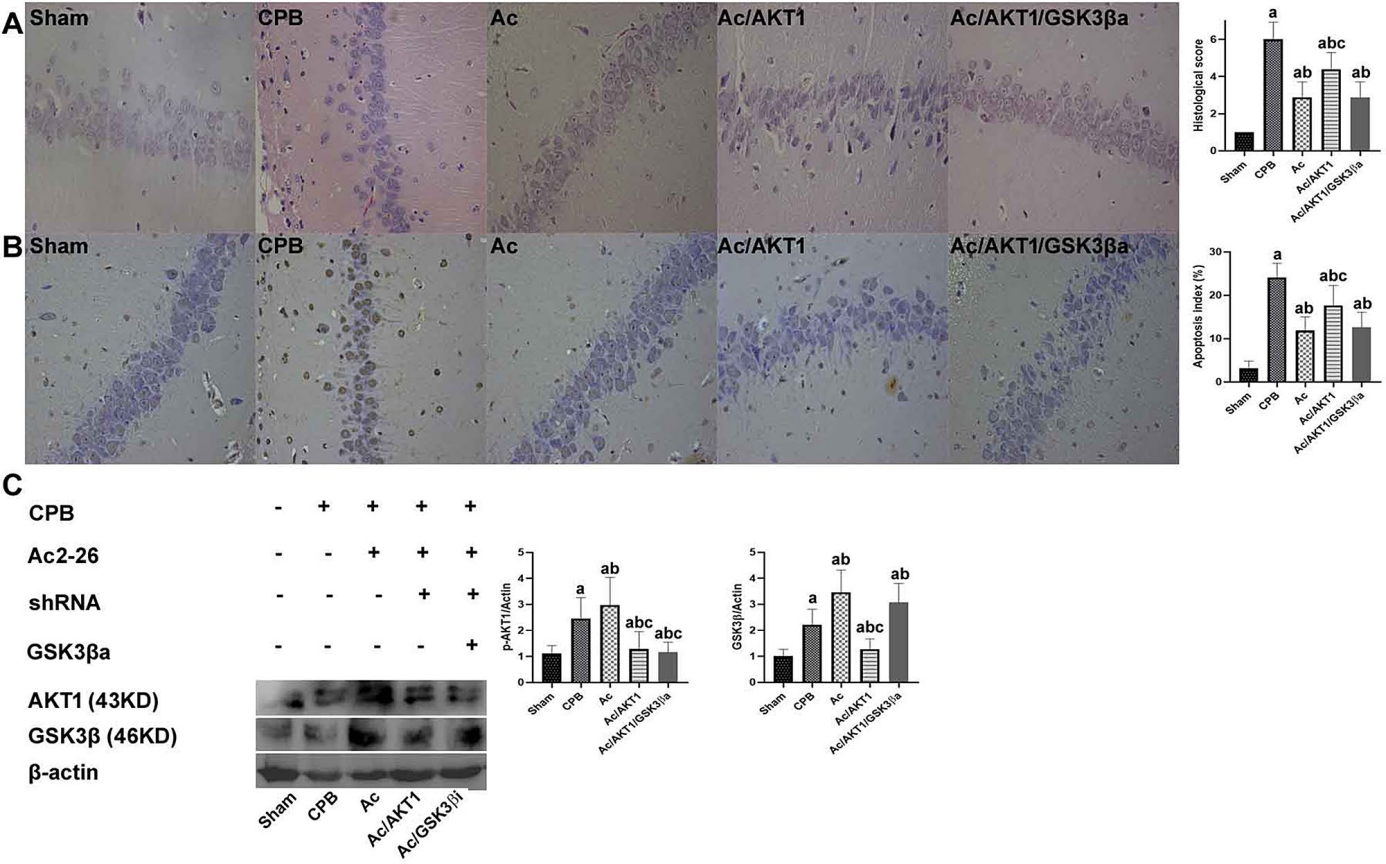
Ac 2–26 inhibited oxidative stress response. *legends*: After 12 h of CPB, the treatment of Ac2-26 on brain damage was totally regained by the agonist of GSK3β (Fig. 7A). Like pathological evaluation, the shRNA significantly lessened the anti-pyroptotic effect of Ac2-26 in Ac/AKT1 group, but the agonist of GSK3β almost recovered by biological effect of Ac2-26 (Fig. 7B). After CPB, the phosphorylation of AKT1 and GSK3β in brain was up-regulated by the Ac2-26. However, the promotion of Ac2-26 on phosphorylation of AKT1 and GSK3β was notably reduced by the shRNA. The agonist of GSK3β did not influence the phosphorylation of AKT1, but notably recovered the phosphorylation of GSK3β. (Fig. 7C)

To investigate the mechanism of Ac2-26 on pyroptosis induced by CPB, we administrated the agonist of GSK3β [26] (Wortmannin 15 µg/kg [27]) to rats received shRNA and inhibitor of GSK3β to rats received Ac2-26. After CPB, the AKT1 expression in brain was down-regulated, but up-regulated by the Ac2-26. The shRNA significantly decreased the expression of AKT1 even underwent stimulation of Ac2-26. The inhibitor of GSK3β did not affect the expression of AKT1. We also found the phosphorylation of GSK3β was inhibited after CPB in brain tissue. The Ac2-26 promoted the phosphorylation of GSK3β after CPB. However, the promotion of Ac2-26 on GSK3β was notably reduced by the shRNA (Fig. 7A). The images were presented with 400 magnifications.

We also tested the pyroptosis in brain tissue. The protection of Ac2-26 on pyroptosis was mainly lessened by the shRNA, but almost reversed by the agonist of GSK3β (Fig. 7B).

## Discussion

The results of the present study indicated that Ac2-26 mitigated the pathological injury and improved cerebral function after CPB via inhibition of inflammation, improvement of brain edema, and regulation of pyroptotic proteins. The protection of Ac2-26 on brain injury after CPB was associated with promotion of AKT1/GSK3β.

CPB is an essential life supporting technique for cardiac surgery. However, CPB induces systemic inflammation and cerebral ischemia reperfusion would lead to severe brain injury, which is characterized by postoperative cognitive dysfunction, delirium, and stroke [12, 28]. Postoperative brain injury after CPB is strongly associated with short- and long-term outcomes [7], such as prolonged ICU and hospital stays, morbidity and mortality [2, 29]. It had been indicated that 30–80% of discharge patients were diagnosed POCD, even 20–40% of patients experienced cognitive impairment for postoperative 6 months [30]. Therefore, effective treatment for brain injury is important for patients underwent CPB.

In this study, we first examined the neurological score, brain wet/dry weight ratio and histological score to estimate the effect of Ac2-26 on brain injury after CPB. The neurological score, brain wet/dry weight ratio and histological score were deteriorated after CPB and Ac2-26 improved the neurological score and brain blood–brain barrier, and pathological score. These results indicated that CPB induced severe cerebral dysfunction, brain edema and brain injury and Ac2-26 significantly improved cerebral function and brain blood–brain barrier, and reduced brain injury after CPB. To further assess the effect of Ac2-26 on brain injury, we also detected the levels of S100β and NSE in serum. S100β and NSE were frequently identified as biomarkers of brain injury. S100β is secreted by mature astroglia cells and Schwann cells, and plays critical role in neuronal survival, neurite extension, axonal proliferation and Ca^2+^-fluxes [31]. Similar to S100β, as a glycolytic enzyme, NSE is mainly presented in neuronal and neuroendocrine tissues, and promotes the conversion of 2-phosphoglycerate to phosphoenolpyruvate. During neuron pathological process, NSE is released into peripheral blood and usually considered to be biomarker of brain injury [28]. Ashraf et al. [35] found that IL-6 strongly promoted the expression of S100-𝛽 during the CPB procedure [32]. When the local or systemic inflammation disrupts the blood-brain barrier, significant S100-𝛽 and NSE is released into peripheral blood. Therefore, the levels of S100-𝛽 and NSE in peripheral blood are sensitive to brain injury [33] and are generally regarded as specific markers of brain injury [34, 35]. In this study, the Ac2-26 notably decreased the levels of S100β and NSE in brain tissue and serum. The results of S100β and NSE were further suggested that Ac2-26 could attenuated the cerebral injury and improved neurological function. These results suggested that Ac2-26 notably reduced brain injury after CPB, which is consistent with previous studies [15, 36].

Although the exact mechanism of postoperative brain injury after CPB is not clear, local ischemia reperfusion injury and SIRS are identified to be major factors for brain injury after CPB [10, 11]. During CPB, contact of blood with artificial surfaces activates proinflammatory cells, and these cells further release inflammatory cytokines, including TNF-α, IL-1β, and IL-6. These cytokines infiltrate into the blood‒brain barrier and damage brain tissue [37]. During CPB, the activated microglia of brain tissue also produces many cytokines that contribute to brain injury in combination with SIRS [15]. The severity of inflammation was strongly associated with the expression of S100-𝛽 protein and NSE. Therefore, we postulated that the protective effect of Ac2-26 on brain injury may be associated with anti-inflammation of Ac2-26. The present study detected the concentrations of inflammatory factors in the brain and serum. We found that Ac2-26 down-regulated the proinflammatory factors, including TNF-α, IL-1β, and IL-6, and up-regulated the anti-inflammatory factor IL-10. These results are consistent with previous studies [15, 18]. The anti-inflammatory effect of Ac2-26 was mainly attributed to the inhibition of activity of NF-κB [13, 18]. During CPB, the NF-κB of inflammatory cells was activated and promoted the secretion of inflammatory factors into brain tissue and the peripheral blood, which ultimately contributed to local and systemic inflammation. In this study, we found that Ac2-26, inhibited the activity of NF-κB [13, 15, 18] and reduced the production of cytokines. The anti-inflammatory effect of Ac2-26 was also associated with the promotion of IL-10. As an important anti-inflammatory factor, IL-10 inhibits the synthesis of proinflammatory cytokines, including TNF-α, IL-1β and IL-6 [38].

During CPB, the brain tissue generally undergoes lower perfusion [10], which results in an oxidative stress response and activation of neutrophils contributed to oxidative stress response. The anaerobic metabolism of neuron and inflammatory cells during CPB would activate the XO, and as a major source of free radicals, the activated XO promoted release of reactive oxygen species (ROS) during the reoxygenation in brain tissue. Moreover, the activated neutrophils during CPB would infiltrate into brain tissue and produced the ROS and resulted in brain damage [39, 40]. During CPB, MPO released from activated microglia and astrocytes in hypoxemia brain tissue, resulting in an increase of reactive oxidants and free radicals and injured the brain tissue. In addition, the ROS not only damaged the cell, but also further led to lipid peroxidation, and finally induced the produce of MDA, which is the final product of lipid peroxidation [24]. In this study, we found the Ac2-26 significantly reduced the MDA levels in brain tissue, which represented the Ac2-26 inhibited severity of oxidative stress response [41]. The anti-oxidant effect of Ac2-26 is associated with the inhibiting of Ac2-26 on activity of XO and MPO. Ac2-26 had been reported to mitigated the inflammation by inhibiting the migration of neutrophils [42] and promoting apoptosis of neutrophils [43]. Therefore, these results demonstrated that Ac2-26 protected brain injury after CPB also partly depended on the anti-oxidative effect.

During CPB, the neuronal cell death induced by inflammatory factors and reactive oxygen species influenced the outcome of animals [15, 19]. As a new programmed cell death mechanism, pyroptosis had been indicated to play critical role in different brain injury [44–46]. However, there was no study investigate the role of pyroptosis in pathology of brain injury after CPB. In this study, we firstly examined the expression of cleaved-GSDMD in brain tissue using immunohistochemistry. We found the positive cells were significantly increased after CPB in brain tissue. This result implied that pyroptosis may be involved in pathology of brain injury after CPB. The cell swelling, plasma membrane pores formation and extravasation of IL-1, IL-18 and lactate dehydrogenase (LDH) are considered to be pathogenic symptoms of pyroptosis [47]. In present study, the LDH, IL-1 and IL-18 in brain tissue were also increased in brain tissue, and these results further implicated that cerebral pyroptosis played important role in brain injury after CPB.

Compared with CPB group, the Ac2-26 significantly decreased the positive cells of cleaved-GSDMD and reduced the levels of LDH, IL-1 and IL-18. These results indicated that Ac2-26 notably inhibited neuronal pyroptosis after CPB. According to previous studies, the NLRP3 inflammasome and precursor of caspase-1 (pro‐caspase‐1), plays an essential role in the initial phase of pyroptosis [48]. Under activation of NLRP3, the activated caspase‐1 is cleaved, and then the cleaved caspase 1 further cleaved the GSDMD, and finally resulting in the formation of membrane pores and the release of IL‐1β and IL‐18 [47, 49]. In this study, we found that Ac2-26 significantly lessened the expression of NLRP3, cleaved-caspase1 and cleaved-GSDMD. These results suggested that the protection of Ac2-26 on brain injury induced by CPB may be associated with regulation of pyroptosis.

Our previous studies demonstrated that organic protection of Ac2-26 was associated with the promotion of eNOS [17, 18]. Considering the main promotion of AKT1 on eNOS [50], and cellular proliferation, migration, and survival of the GSK3β, which is important down-stream of AKT1, we assumed that Ac2-26 presented cerebral protection in CPB via promotion of AKT1/GSK3β pathway. To certify this postulation, we administrated shRNA to knock-down the AKT1 and inhibitor or agonist of GSK3β to rats received Ac2-26. We found that shRNA and inhibitor of GSK3β notably weakened the protection of Ac2-26. However, in the rats received shRNA, the agonist of GSK3β almost recovered the protective effect of Ac2-26 on brain injury. This result further suggested that therapy of Ac2-26 on brain injury partly depended on promotion of AKT1/GSK3β.

The Annexin A1 is an endogenous and glucocorticoid-inducible anti-inflammatory protein, had been demonstrated to protect against multiple organ injury by regulating inflammation, cellular death and modulation of inflammatory cells, but avert adverse effect of glucocorticoids. Therefore, the Annexin 1 or it’s activator peptide Ac2-26 provided a new insight or strategy for treatment of brain injury after CPB in clinical work.

### Limitation

There were some limitations in this study. The first is that we did not observe the long-term outcome of rat after CPB. In our future study, we will estimate the long-term outcome of Ac2-26 on cerebral injury after CPB, especially recognitive function. The second is the deeply mechanism of Ac2-26 on AKT1/GSK3β pathway. In our further study, we will apply the single-cell sequencing analysis to investigate the exactly mechanism of Ac2-26 on AKT1. The third is the conclusion of Ac2-26 on cerebral pyroptosis was based on the pyroptotic protein from brain tissue. We will deeply detect the effect of Ac2-26 on cerebral cell and inflammatory cells in our future study.

## Conclusion

Based on these results, we concluded that Ac2-26 reduced CPB-induced neuron pyroptosis, improved cerebral function and pathological injury via inhibition of inflammation and oxidative stress response. The protective effect of Ac2-26 primarily depended on the promotion of AKT1/GSK3β.

### Humane endpoints

According to the guidelines of the University of California, Berkeley, all rats undergoing surgery must be monitored for humane endpoints every four hours postoperatively. The humane endpoints including:

1. Observe the behavior and posture of the rats: Check for any abnormal symptoms such as pain, difficulty breathing, or inability to eat or drink. Prompt action should be taken if these symptoms are observed to alleviate their suffering.
2. Examine wounds and surgical sites: Look for signs of infection, bleeding, or swelling at the surgical site. If these issues are found, they need to be addressed promptly to prevent further deterioration.
3. Monitor life signs: This includes temperature, heart rate, and respiratory rate. These indicators can reflect the overall condition of the rats, and any abnormalities require prompt attention.
4. Record monitoring results: After each monitoring session, detailed notes should be taken on the condition of the rats, including appetite, activity level, and wound status. These records can be used to assess the health status of the rats and to promptly identify and address any issues.

### Electronic supplementary material

Below is the link to the electronic supplementary material.

Supplementary Material 1

Supplementary Material 2

## Data Availability

The authors confirm that the data supporting the findings of this study are available within the article and its supplementary materials.
