# Supplementary material for: Ac2-26 activated the AKT1/GSK3β pathway to reduce cerebral neurons pyroptosis and improve cerebral function in rats after cardiopulmonary bypass
Source: BMC Cardiovasc Disord. 2024 May 21;24:266. doi: 10.1186/s12872-024-03909-9 (PMC11106860; doi:10.1186/s12872-024-03909-9)
Supplement: Supplementary file 1 — Supplementary Material 1 [file 12872_2024_3909_MOESM1_ESM.docx]

raw figure


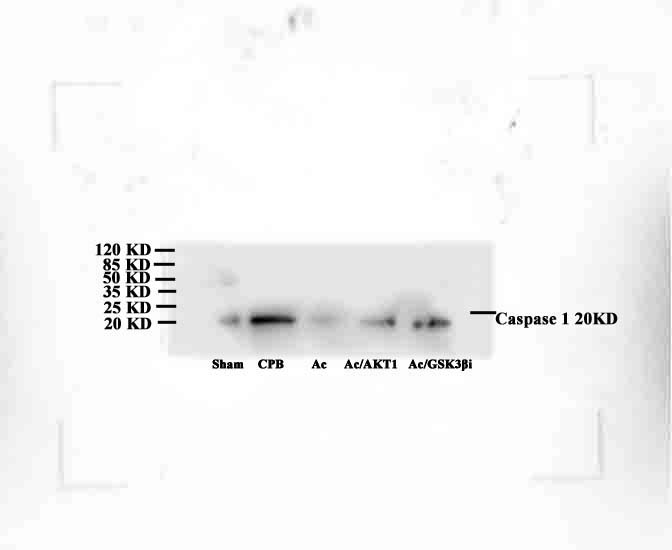


Figure 4 caspase 1.jpg


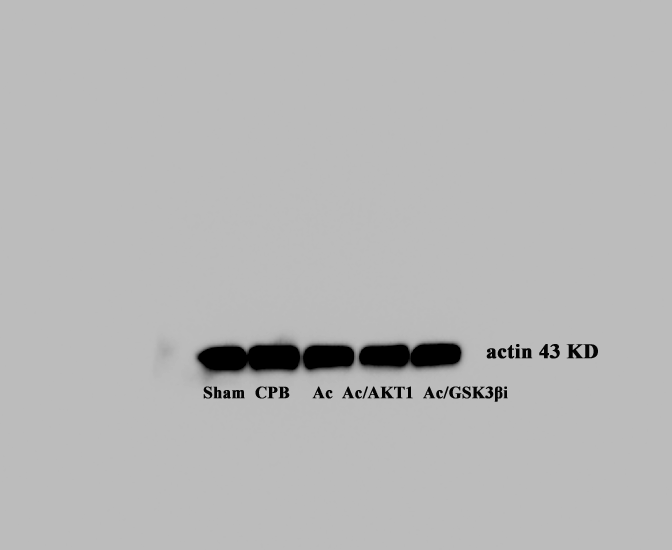


Figure 4 actin.tif


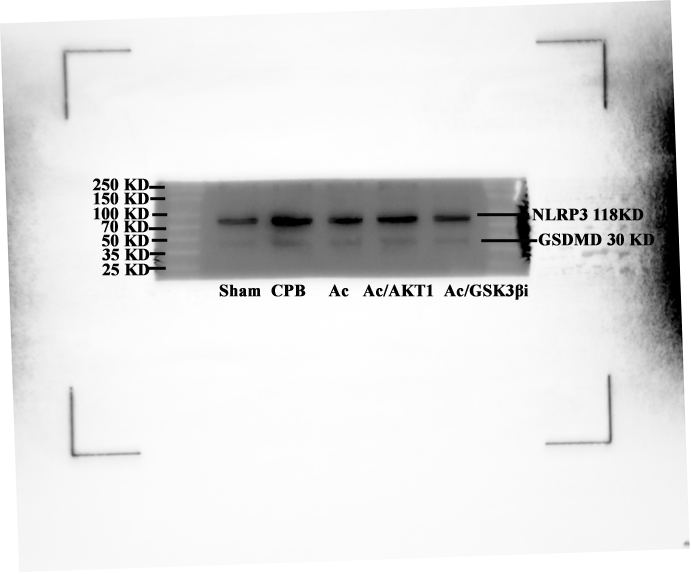


Figure 4 NLRP3 and GSDMD.tif


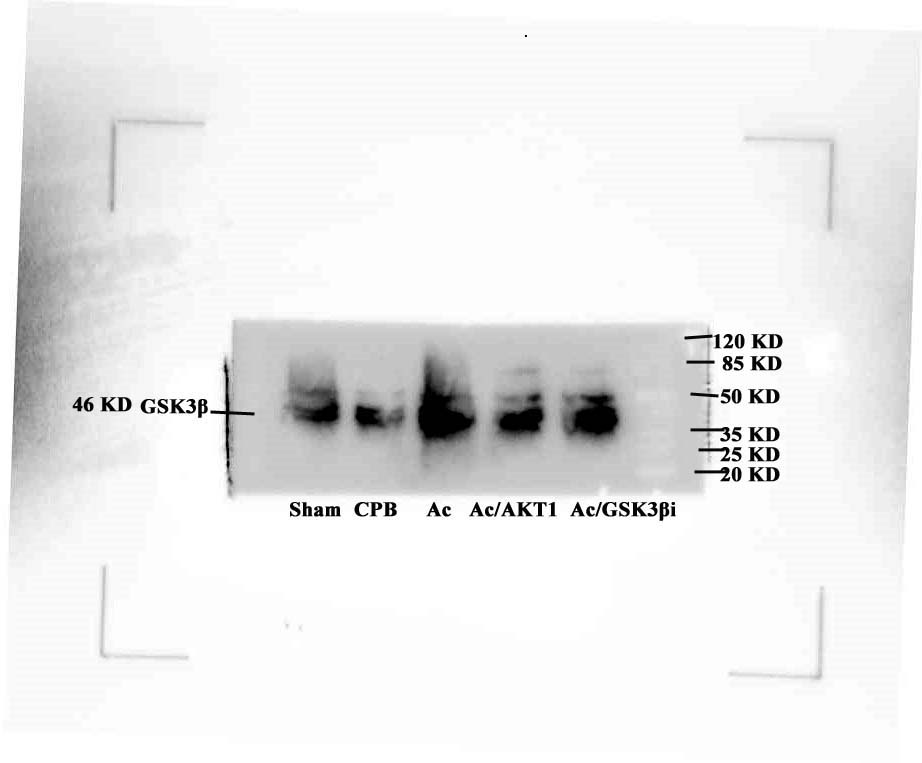


Figure 7 GSK 3 beta.jpg


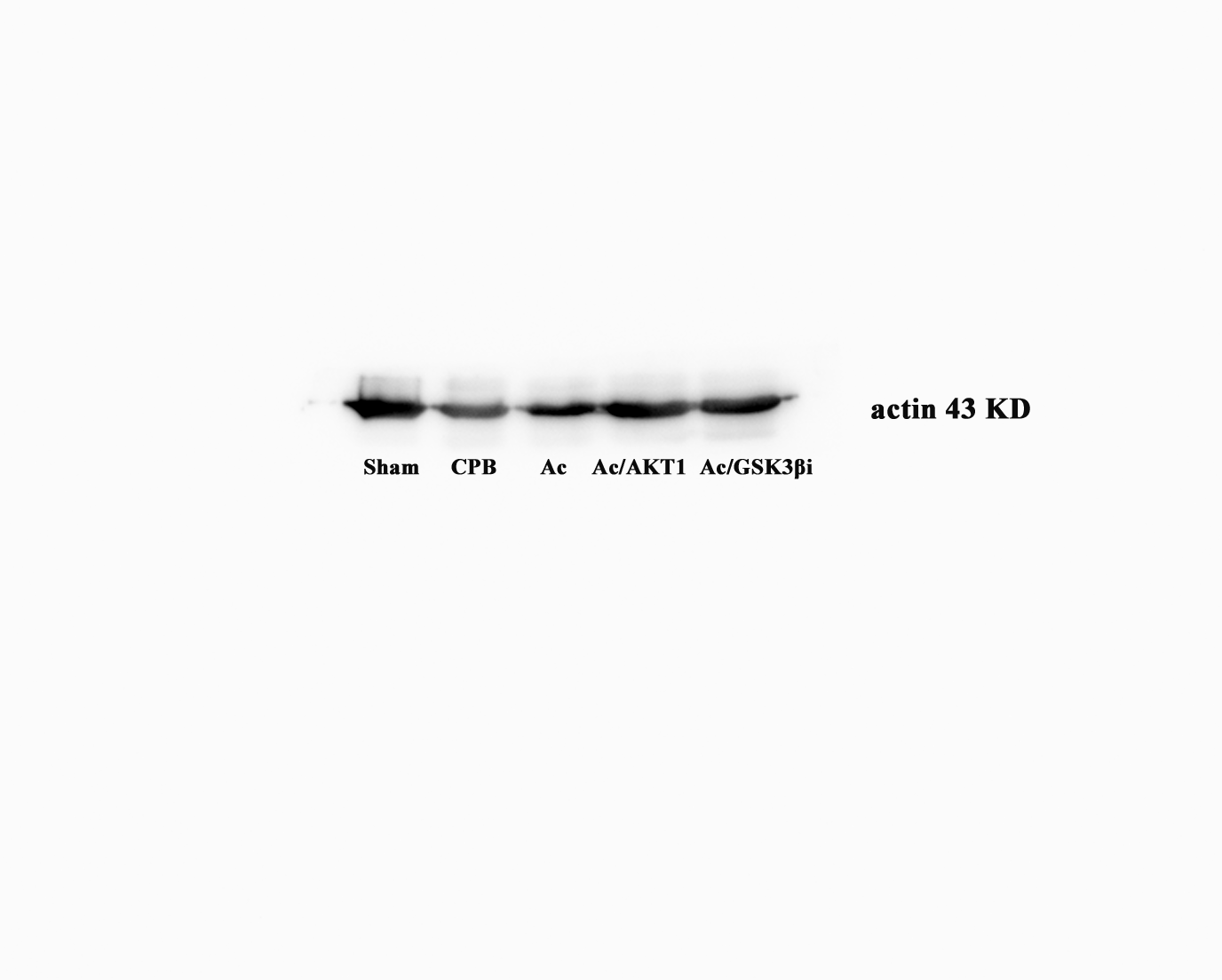


Figure 7 actin.tif


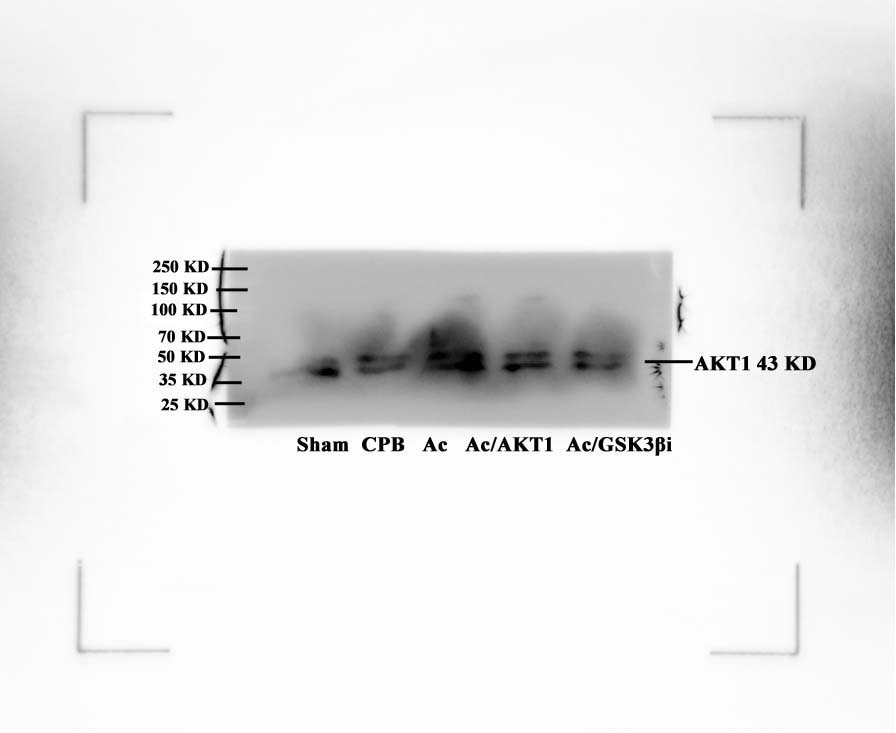


Figure 7 AKT1 拷贝.jpg
