# Supplementary material for: Ac2-26 activated the AKT1/GSK3β pathway to reduce cerebral neurons pyroptosis and improve cerebral function in rats after cardiopulmonary bypass
Source: BMC Cardiovasc Disord. 2024 May 21;24:266. doi: 10.1186/s12872-024-03909-9 (PMC11106860; doi:10.1186/s12872-024-03909-9)

Sham	CPB	Ac	Ac/AKT1	Ac/AKT1/GSK3βa
0.61	2.64	3.83	1.05	1.25
1.19	2.38	2.52	0.74	0.94
0.74	3.05	3.89	1.69	0.68
1.34	2.27	4.26	0.86	1.14
1.53	1.02	2.15	0.93	1.38
1.27	2.36	3.69	1.07	1.42
1.21	2.13	1.37	1.14	0.72
0.99	3.83	2.06	2.79	1.81

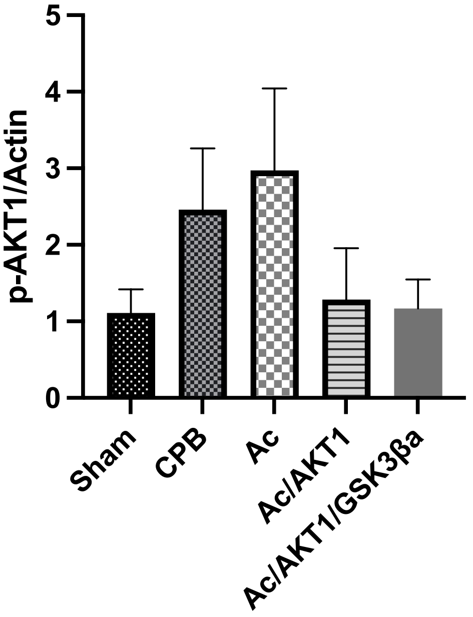

Sham	CPB	Ac	Ac/AKT1	Ac/GSK3βi
1	5	3	4	5
1	6	2	5	6
1	5	3	4	5
1	5	4	6	4
2	7	3	4	5
1	6	4	5	3
1	5	2	4	4
1	7	3	3	3

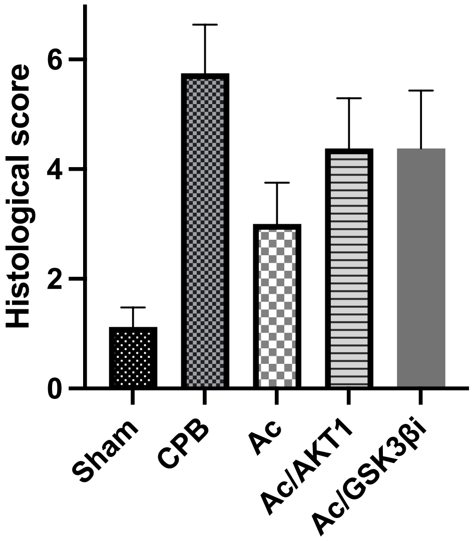

Sham	CPB	Ac	Ac/AKT1	Ac/GSK3βi
0.56	0.94	0.68	0.76	0.68
0.35	0.83	0.71	0.63	0.86
0.57	0.96	0.59	0.92	0.77
0.38	0.83	0.65	0.69	0.92
0.39	0.96	0.58	0.68	1.01
0.46	0.78	0.46	0.93	0.81
0.43	0.97	0.55	0.78	0.73
0.61	1.12	0.65	0.82	0.52

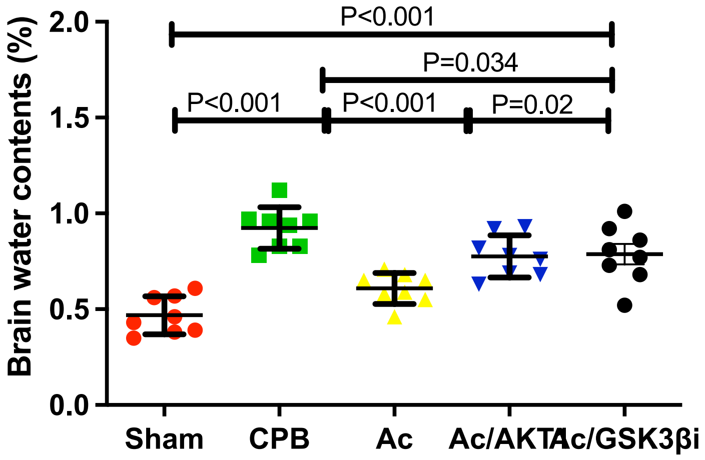

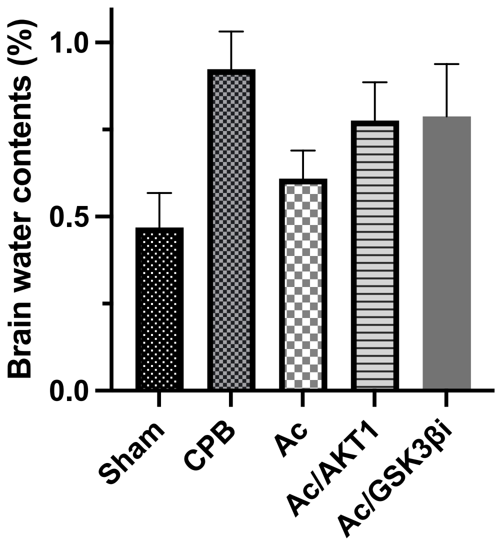

Sham	CPB	Ac	Ac/AKT1	Ac/GSK3βi
0.87	3.27	1.83	2.87	2.55
0.31	3.34	2.2	2.34	2.18
0.65	3.1	2.8	2.1	3.04
1.1	2.78	2.16	3.88	2.97
1.09	3.15	1.66	2.15	2.81
1.03	4.06	1.44	3.06	3.35
1.04	3.94	2.51	2.94	2.24
0.81	3.6	2.08	2.6	2.73

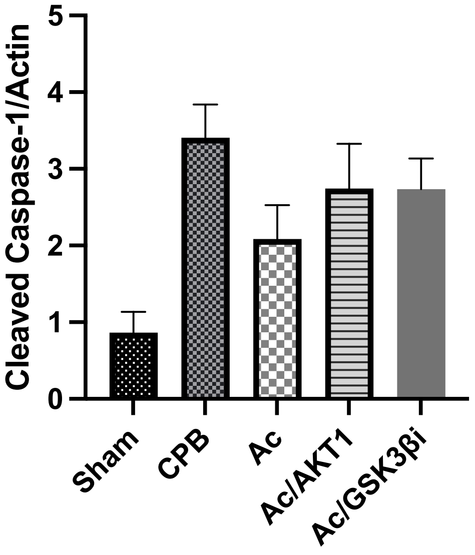

Sham	CPB	Ac	Ac/AKT1	Ac/GSK3βi
0	5	3	4	3
1	4	3	3	4
0	6	2	5	5
0	5	2	4	3
1	5	3	3	4
0	6	3	4	4
0	4	2	5	4
1	5	4	3	4

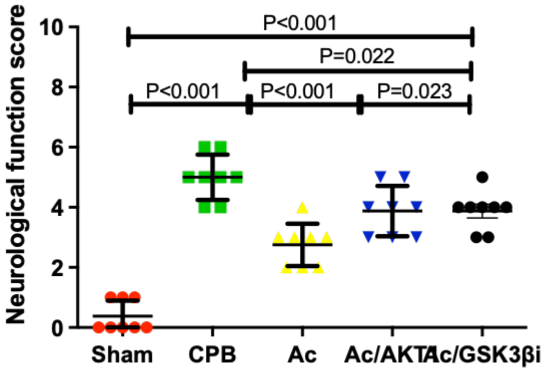

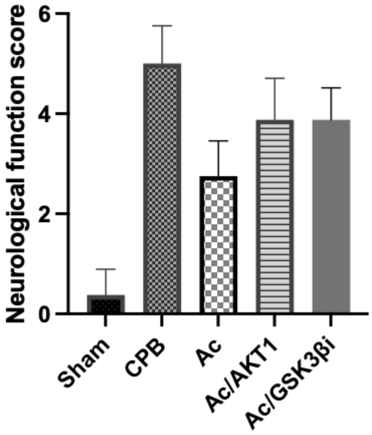

Sham	CPB	Ac	Ac/AKT1	Ac/GSK3βi
0.82	2.13	4.07	1.22	1.28
1.06	3.12	2.33	0.98	1.08
0.53	2.28	3.94	1.24	1.15
1.04	2.41	3.61	0.21	0.93
0.96	1.37	3.72	1.76	1.18
0.81	2.26	2.56	0.83	1.52
0.73	2.95	3.33	1.41	0.88
0.88	1.68	2.78	1.17	1.33

Sham	CPB	Ac	Ac/AKT1	Ac/GSK3βi
1.37	3.97	2.53	3.22	3.31
1.26	4.23	3.12	2.98	3.59
0.53	4.1	2.42	2.74	3.76
1.51	3.06	2.13	3.21	4.11
1.06	5.2	1.17	3.76	3.18
1.11	4.56	2.06	4.13	2.52
1.13	3.73	2.61	3.41	3.36
1.08	4.8	1.38	3.7	3.68

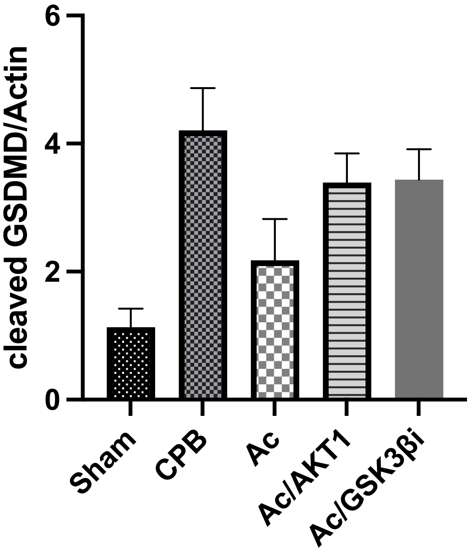

Sham	CPB	Ac	Ac/AKT1	Ac/AKT1/GSK3βa
0.62	1.48	4.82	1.29	2.25
1.4	2.09	2.98	0.73	4.11
0.92	2.24	3.51	0.98	2.22
1.18	3.17	2.85	1.25	3.43
0.83	1.82	3.83	1.47	2.98
0.79	1.77	4.07	1.31	3.77
1.25	3.02	3.62	2.06	2.48
1.06	2.13	1.99	1.14	3.45

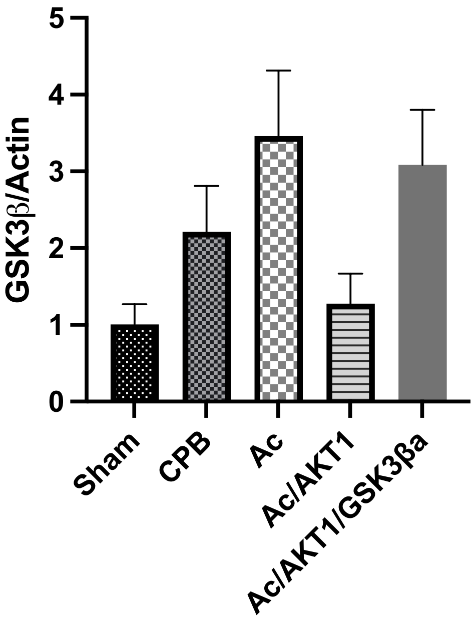

Sham	CPB	Ac	Ac/AKT1	Ac/GSK3βi
12.6	31.8	24.8	29.7	32.5
15.4	30.9	19.1	33.7	29.6
9.2	39.8	25.6	32.9	25.1
11.8	34.4	21.5	28.5	30.4
10.3	40.2	18.3	27.8	27.8
10.9	29.7	30.2	35.1	24.2
11.5	36.2	23.6	30.6	28.7
9.6	50.3	19.5	26.4	31.4

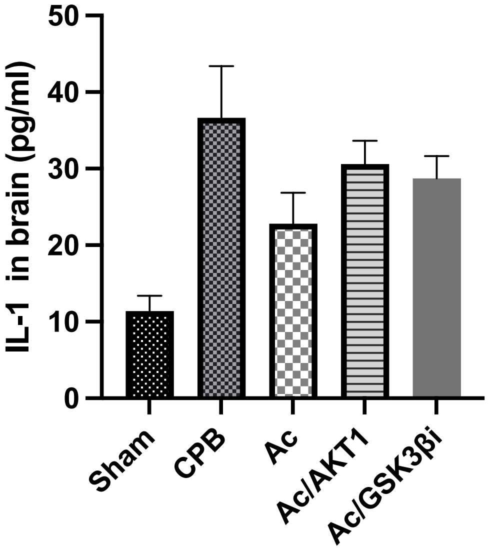

Sham	CPB	Ac	Ac/AKT1	Ac/GSK3βi	Sham	CPB	Ac	Ac/AK
89.6	90.7	114.5	98.3	79.7	114.5	272.4	209.4	18
104.9	111.7	108.2	107.9	113.6	108.2	259.9	194.7	22
145.9	152.5	80.7	82.7	137.1	90.7	229.2	171.3	25
108.3	112.3	108.1	116.8	98.5	108.1	246.5	215.5	21
95.6	100.9	121.3	103.2	105.4	121.3	308.7	201.8	21
123.3	129.8	99.2	112.4	122.6	99.2	221.8	165.2	24
117.81	122.4	119.8	126.5	89.3	109.8	237.2	208.4	23
76.5	88.3	106.8	112.6	100.8	81.2	291.4	176.3	24

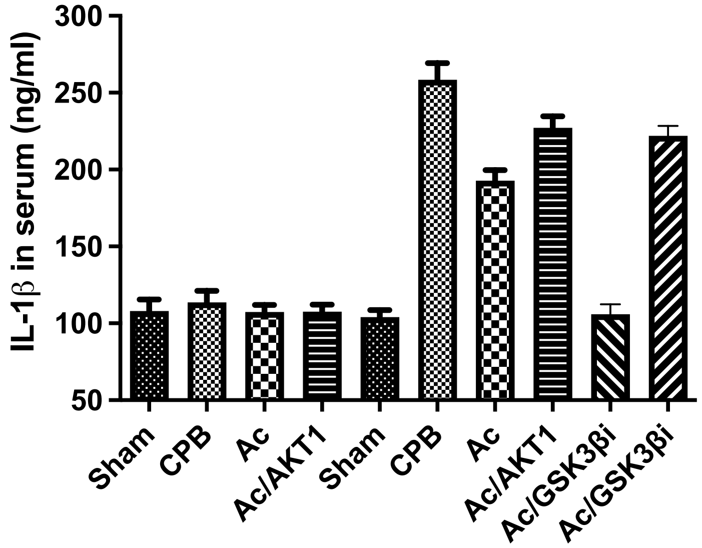

		Sham			CPB			Ac			Ac/AKT1
Baseline	107.7388	21.58194	8	113.575	21.32797	8	107.325	12.97776	8	107.55	13.1791
12 hours after CPB	104.125	13.0782	8	258.3875	30.70735	8	192.825	19.32827	8	227.275	20.70471

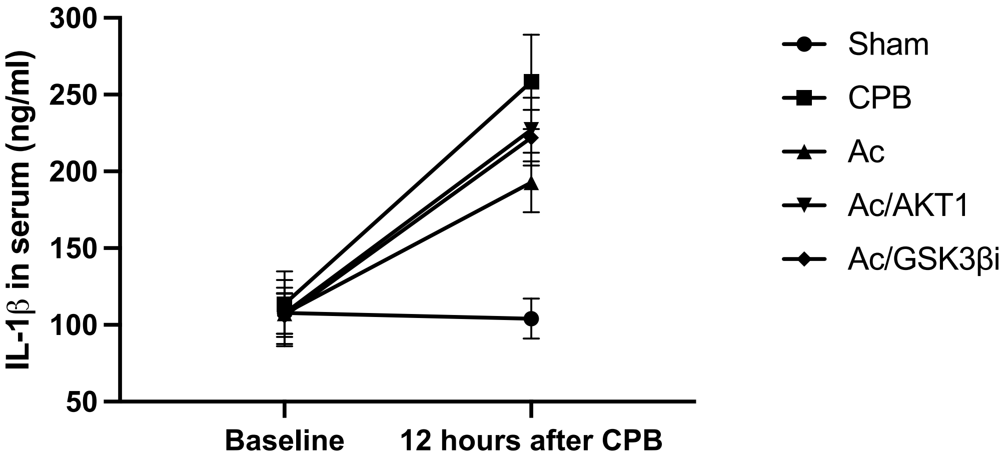

Sham	CPB	Ac	Ac/AKT1	Ac/GSK3βi
30.6	54.3	33.1	49.6	45.9
28.9	65.2	38.2	53.1	57.6
24.1	51.7	41.3	48.4	44.3
29.4	64.5	46.5	65.3	50.8
26.3	73.3	32.8	39.5	47.9
32.3	59.4	41.7	47.5	35.2
21.2	68.8	38.4	56.4	48.8
31.8	56.4	36.3	52.2	66.4

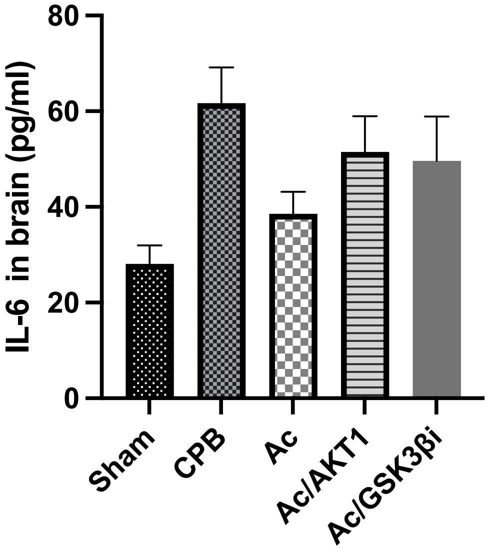

Sham	CPB	Ac	Ac/AKT1	Ac/GSK3βi	Sham	CPB	Ac	Ac/AKT
7.6	7.9	11.7	6.8	9.2	8.2	39.7	19.5	
8.4	10.6	10.9	9.4	6.7	9.3	39.6	20.3	
10.8	11.3	13.4	5.2	5.8	11.1	57.2	9.9	
6.7	7.8	6.8	10.7	10.1	7.1	35.1	15	
9.2	10.5	7.9	8.3	9.5	8.5	41.9	26.2	
11.3	8.2	8.6	10.2	8.4	9.2	34.8	25.2	
10.1	9.2	10.1	6.5	12.3	11.4	52.8	11.7	
8.8	10.3	9.4	5.9	7.4	9.2	31.3	9.5	

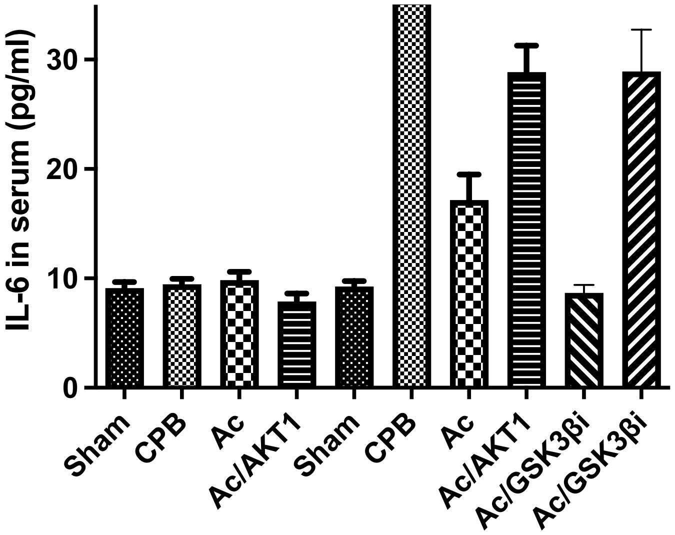

	Sham			CPB			Ac			Ac/AKT1		
Baseline	9.1125	1.573384	8	9.475	1.379182	8	9.85	2.140093	8	7.875	2.070024	8.675
12 hours after CPB	9.25	1.431283	8	41.55	9.032165	8	17.1625	6.628496	8	28.8625	6.866053	28.9125

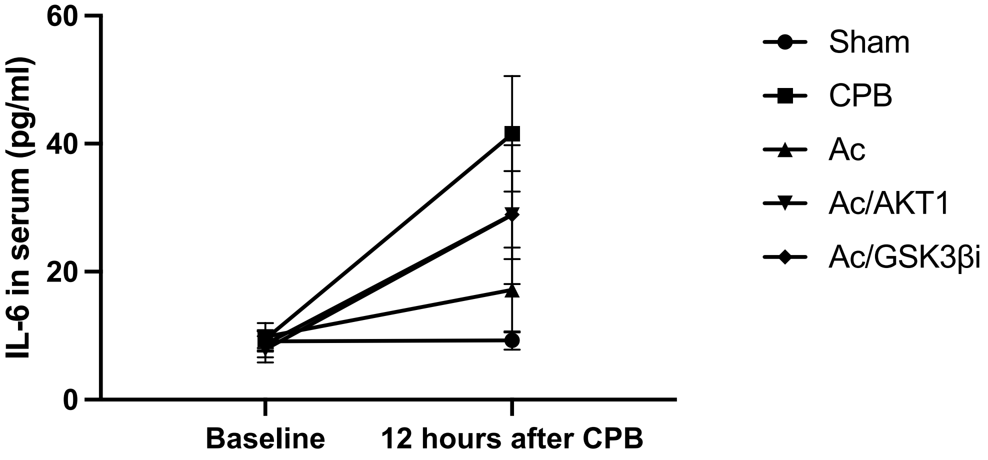

Sham	CPB	Ac	Ac/AKT1	Ac/GSK3βi
9.6	19.8	31.8	29.7	23.8
8.4	11.1	34.9	25.7	31.1
11.2	17.6	22.8	19.9	14.6
10.7	13.5	43.4	11.5	13.3
12.3	16.3	30.2	17.8	17.4
9.1	14.2	26.7	27.1	15.7
10.9	21.6	21.2	15.6	20.8
13.3	10.7	30.3	23.4	26.5

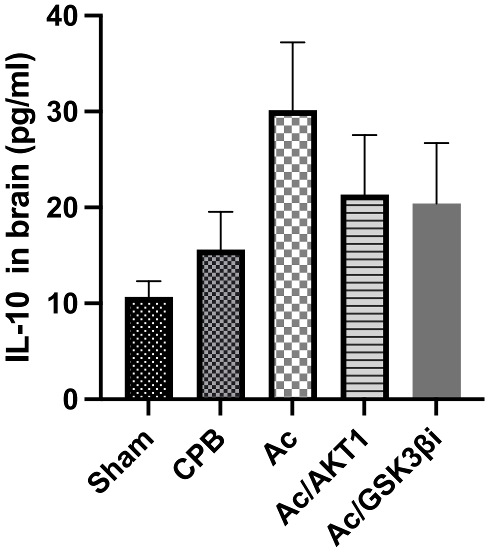

Sham	CPB	Ac	Ac/AKT1	Ac/GSK3βi
67.6	301.1	228.3	267.1	245.8
74.7	332.3	197.7	223.3	277.6
47.1	289.5	256.2	249.7	235.3
57.9	316.7	215.5	275.8	300.3
44.3	294.7	187.1	278.4	246.8
87.1	277.6	210.82	301.6	222.7
60.5	315.9	206.4	256.5	237.2
89.6	253.5	195.4	180.6	253.4

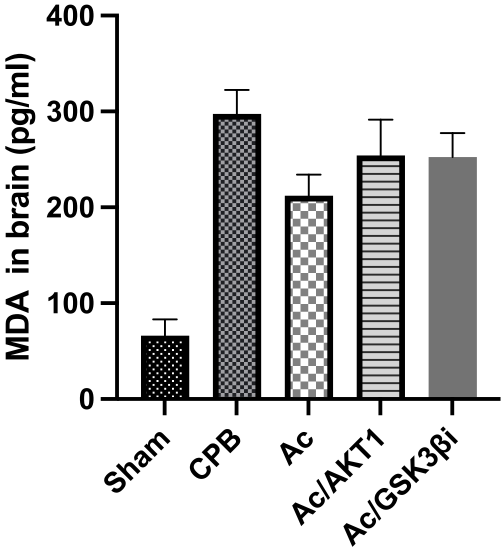

Sham	CPB	Ac	Ac/AKT1	Ac/GSK3βi
1.2	5.2	2.2	2.8	2.1
0.4	6.1	1.1	3.8	3.5
1.2	4.7	2.5	2.9	4.1
2.8	4.2	0.8	2.6	4.6
1.3	3.2	1.6	3.8	2.7
0.9	4.1	3.1	3.3	3.2
1.5	5.7	1.3	2.9	1.4
0.6	5.3	1.5	4.4	3.7

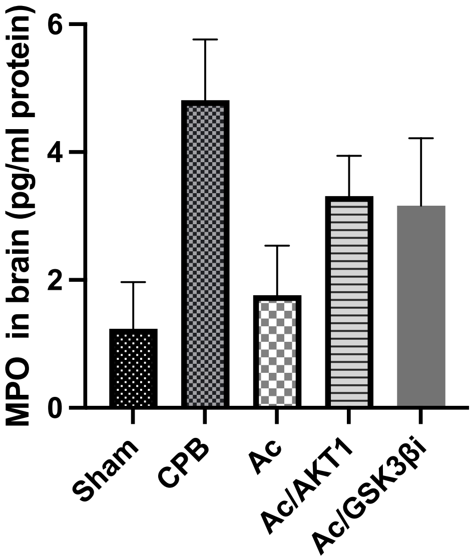

Sham	CPB	Ac	Ac/AKT1	Ac/GSK3βi
0.82	2.43	1.07	2.22	1.57
0.26	2.97	0.56	1.07	2.21
0.09	1.92	1.14	1.47	0.94
0.31	1.22	0.61	1.21	1.29
0.46	2.75	0.72	1.56	1.46
0.21	2.66	0.89	1.07	1.11
0.33	1.84	0.77	1.41	2.55
0.49	3.82	0.68	2.95	2.08

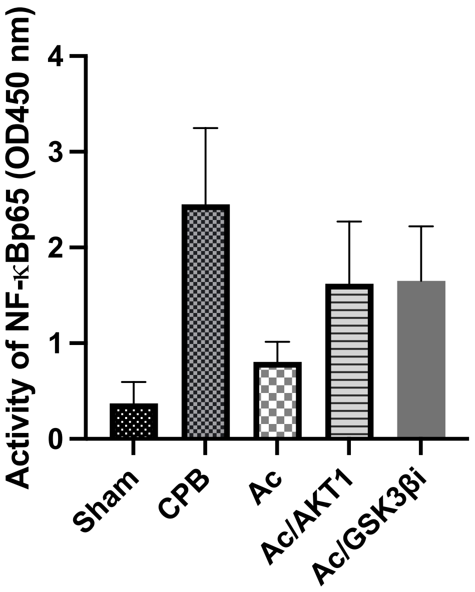

Sham	CPB	Ac	Ac/AKT1	Ac/GSK3βi
0.47	3.71	1.95	2.79	2.59
0.51	4.41	2.62	2.54	3.76
0.78	3.33	1.08	2.31	1.83
0.92	2.36	1.26	3.78	3.38
1.1	3.65	2.68	2.55	1.79
0.84	4.12	1.91	3.16	2.32
1.01	3.48	2.12	2.61	3.44
0.61	3.82	1.48	2.35	2.57

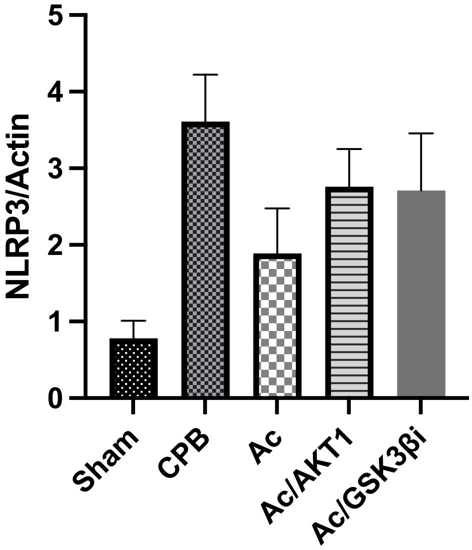

Sham	CPB	Ac	Ac/AKT1	Ac/GSK3βi
5.6	17.8	12.8	16.7	15.8
7.4	18.9	15.1	15.1	14.5
9.2	18.2	11.6	12.9	12.7
6.8	15.4	10.5	18.5	17.2
8.3	20.2	13.3	16.8	17.6
7.7	16.7	16.2	13.3	16.4
6.5	21.2	14.6	17.6	15.3
6.3	20.3	10.7	16.4	15.9

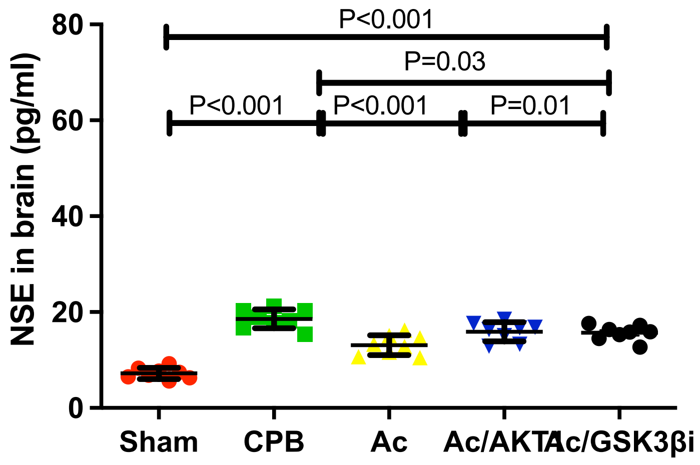

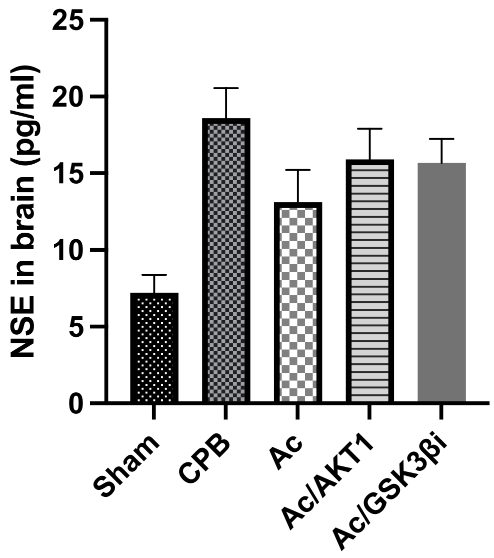

Sham	CPB	Ac	Ac/AKT1	Ac/GSK3βi
2.3	21.2	10.1	14.1	15.7
4.8	26.8	8.5	10.3	19.1
5.2	18.4	13.8	18.6	14.2
1.9	27.6	9.9	20.9	20.4
3.9	19.3	14.5	23.7	9.6
3.4	23.7	8.2	16.8	15.3
4.6	20.4	17.7	20.9	18.8
1.7	25.5	10.3	11.2	24.6

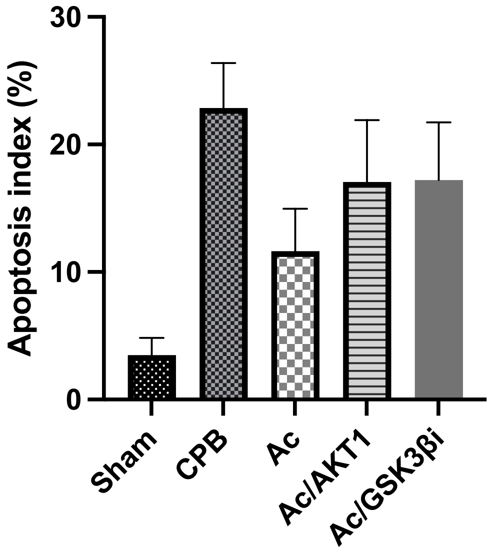

Sham	CPB	Ac	Ac/AKT1	Ac/AKT1/GSK3βa
2.1	23.2	12.5	15.7	11.4
4.4	27.1	8.9	13.6	15.1
5.5	22.5	10.8	15.2	14.2
4.1	23.7	7.2	22.3	13.6
1.2	17.8	17.1	13.2	9.3
2.3	26.3	12.6	26.5	10.3
4.4	23.6	14.7	18.5	18.8
1.1	28.5	11.1	16.2	8.6

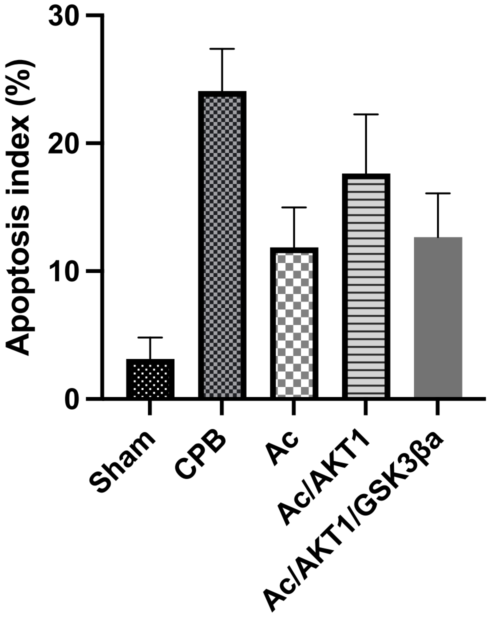

Sham	CPB	Ac	Ac/AKT1	Ac/GSK3βi
0.08	0.58	0.38	0.55	0.47
0.12	0.42	0.41	0.47	0.51
0.07	0.64	0.36	0.53	0.63
0.09	0.76	0.25	0.65	0.38
0.05	0.88	0.39	0.48	0.62
0.07	0.69	0.42	0.46	0.49
0.11	0.68	0.29	0.39	0.45
0.08	0.51	0.37	0.43	0.54

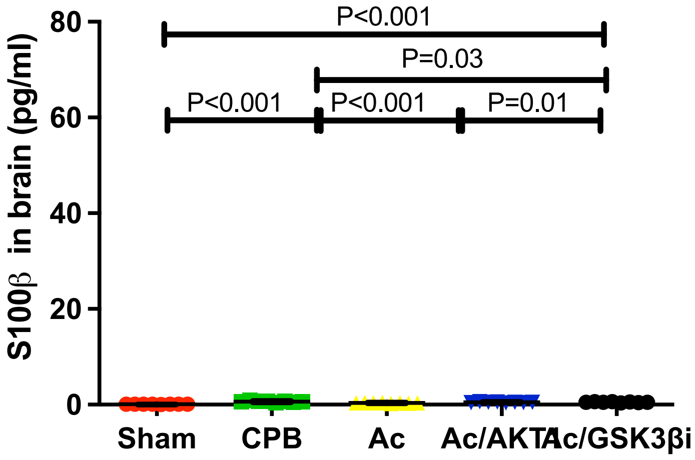

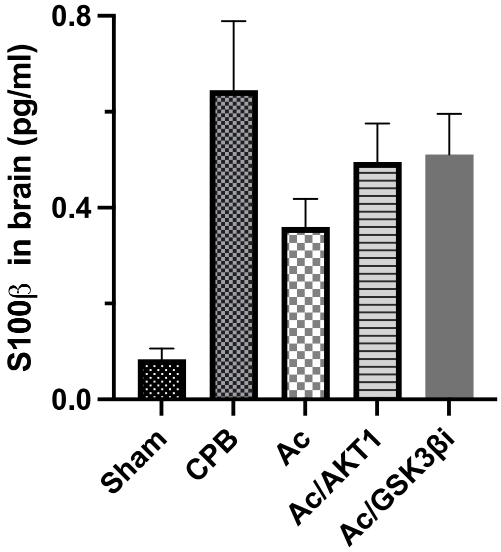

Sham	CPB	Ac	Ac/AKT1	Ac/GSK3βi
100	56.5	87.3	67.1	55.7
100	65.7	79.8	75.3	67.2
100	48.9	76.5	68.6	70.9
98	61.2	83.7	89.9	68.3
100	59.4	79.4	60.7	72.7
99	63.6	85.6	70.8	75.1
97	68.3	80.4	59.9	81.9
100	67.1	88.2	79.2	79.6

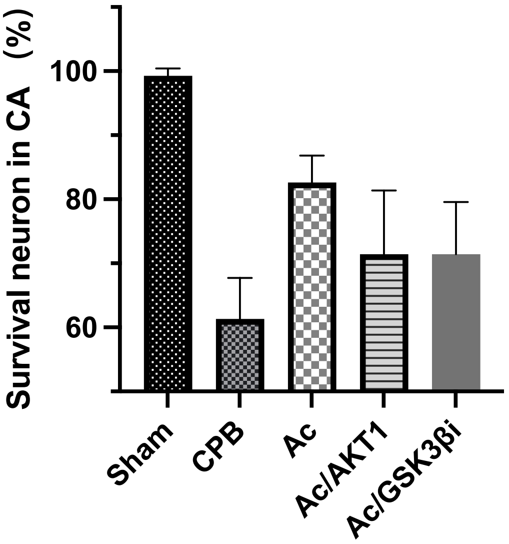

Sham	CPB	Ac	Ac/AKT1	Ac/GSK3βi
96.9	59.3	83.3	73.3	75.2
98.4	65.7	88.8	85.6	67.2
97.7	69.7	92.6	69.1	80.9
98.4	62.6	80.7	77.2	73.1
100	79.1	89.9	84.5	83.4
97.3	71.5	87.6	80.8	79.6
98.2	54.9	92.4	74.9	77.3
95.8	78.4	76.2	70.7	81.8

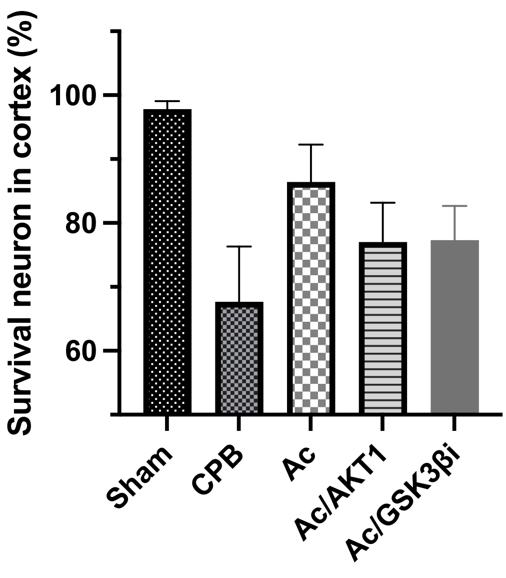
Survival neuron in cortex (%)
100
80
60
Sham
CPB
Ac
Ac/AKT1
Ac/GSK3βi

Sham	CPB	Ac	Ac/AKT1	Ac/GSK3βi
1.4	9.6	5.2	8.8	5.9
2.2	8.9	4.2	5.9	7.6
3.5	10.7	3.3	7.5	4.3
2.9	6.1	5.8	5.4	10.8
1.1	12.3	6.6	8.6	7.9
3.3	8.4	4.2	6.3	5.2
2.5	11.5	5.1	7.2	8.8
1.6	7.8	3.5	8.5	6.4

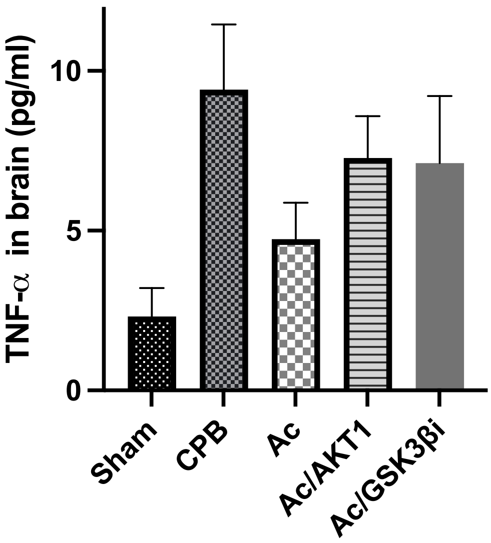

Sham	CPB	Ac	Ac/AKT1	Ac/GSK3βi	Sham	CPB	Ac	Ac/AKT
10.7	5.1	8.8	8.8	13.6	11.1	25.4	13.5	
9.8	13.2	12.7	5.9	6.8	10.5	31.1	18.4	
5.4	8.5	9.4	13.2	9.7	8.6	47.5	25.2	
11.2	7.6	10.3	7.9	8.1	10.9	52.6	19.7	
7.6	15.9	11.6	8.3	11.4	8.7	35.2	21.4	
15.5	8.8	16.9	15.6	14.2	12.9	60.7	23.1	
14.2	10.2	6.2	12.1	7.6	16.1	43.3	18.6	
8.6	7.6	7.5	9.3	10.5	7.4	59.2	22.8	

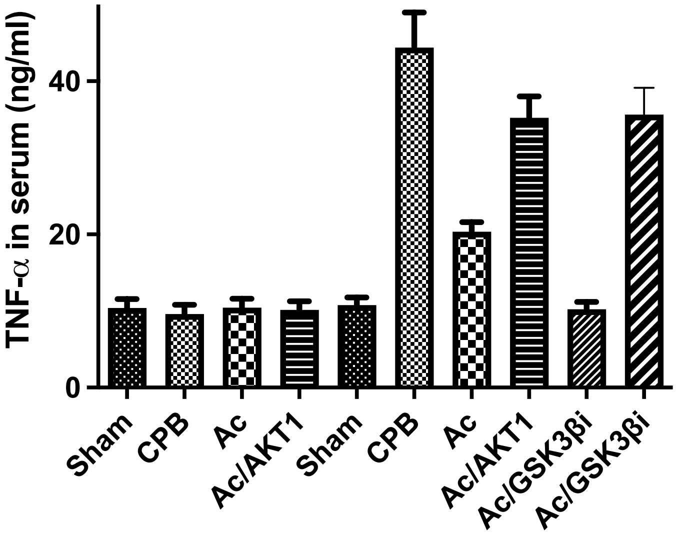

	Sham			CPB			Ac			Ac/AKT1			
Baseline	10.375	3.329843	8	9.6125	3.445675	8	10.425	3.350799	8	10.1375	3.204879	8	10.237
12 hours after CPB	10.775	2.764443	8	44.375	13.01271	8	20.3375	3.633156	8	35.2375	7.86274	8	35.637

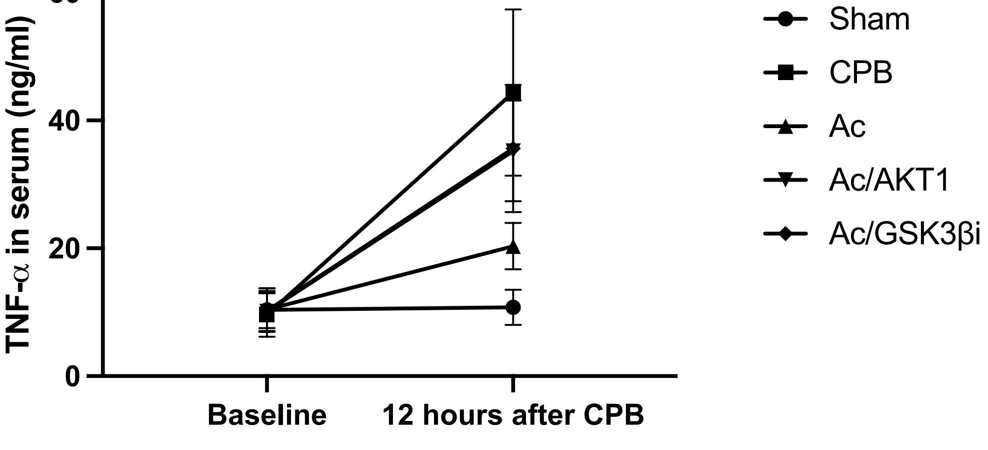

Sham	CPB	Ac	Ac/AKT1	Ac/GSK3βi
15.1	73.1	43.8	67.3	74.5
12.3	90.2	51.9	73.2	58.6
24.5	79.3	55.3	62.5	75.7
18.7	84.8	41.7	58.7	60.9
20.7	82.2	38.8	77.4	77.3
16.2	68.5	50.4	65.8	64.2
21.6	95.6	62.6	70.5	78.8
16.3	73.7	35.4	56.6	51.1

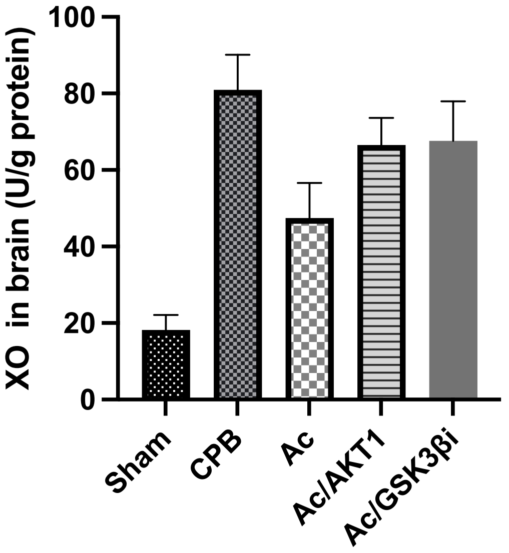

Supplement: Supplementary file 2 — Supplementary Material 2 [file 12872_2024_3909_MOESM2_ESM.pdf]
